# Diversity in Phytochemical Composition, Antioxidant Capacities, and Nutrient Contents Among Mungbean and Lentil Microgreens When Grown at Plain-Altitude Region (Delhi) and High-Altitude Region (Leh-Ladakh), India

**DOI:** 10.3389/fpls.2021.710812

**Published:** 2021-07-30

**Authors:** Priti, Gyan P. Mishra, Harsh K. Dikshit, Vinutha T., M. Tomuilim Tontang, Tsering Stobdan, Seema Sangwan, Muraleedhar Aski, Ajeet Singh, Ranjeet R. Kumar, Kuldeep Tripathi, Shiv Kumar, Ramakrishnan M. Nair, Shelly Praveen

**Affiliations:** ^1^Division of Genetics, Indian Council of Agricultural Research-Indian Agricultural Research Institute, New Delhi, India; ^2^Division of Biochemistry, Indian Council of Agricultural Research-Indian Agricultural Research Institute, New Delhi, India; ^3^Defence Research and Development Organisation-Defence Institute of High Altitude Research, Leh, India; ^4^Division of Genetics, Indian Council of Agricultural Research-Indian Agricultural Research Institute, New Delhi, India; ^5^Germplasm Evaluation Division, Indian Council of Agricultural Research-National Bureau of Plant Genetic Resources, New Delhi, India; ^6^Biodiversity and Integrated Gene Management Program, International Center for Agricultural Research in the Dry Areas, Rabat, Morocco; ^7^World Vegetable Center, South Asia, International Crops Research Institute for the Semi-Arid Tropics Campus Patancheru, Hyderabad, India

**Keywords:** *Vigna* microgreens, *Lens* microgreens, Fabaceae microgreens, antioxidants, mineral composition

## Abstract

Mungbeans and lentils are relatively easily grown and cheaper sources of microgreens, but their phytonutrient diversity is not yet deeply explored. In this study, 20 diverse genotypes each of mungbean and lentil were grown as microgreens under plain-altitude (Delhi) and high-altitude (Leh) conditions, which showed significant genotypic variations for ascorbic acid, tocopherol, carotenoids, flavonoid, total phenolics, DPPH (1, 1-diphenyl-2-picrylhydrazyl), FRAP (ferric-reducing antioxidant power), peroxide activity, proteins, enzymes (peroxidase and catalase), micronutrients, and macronutrients contents. The lentil and mungbean genotypes L830 and MH810, respectively, were found superior for most of the studied parameters over other studied genotypes. Interestingly, for most of the studied parameters, Leh-grown microgreens were found superior to the Delhi-grown microgreens, which could be due to unique environmental conditions of Leh, especially wide temperature amplitude, photosynthetically active radiation (PAR), and UV-B content. In mungbean microgreens, total phenolics content (TPC) was found positively correlated with FRAP and DPPH, while in lentil microgreens, total flavonoid content (TFC) was found positively correlated with DPPH. The most abundant elements recorded were in the order of K, P, and Ca in mungbean microgreens; and K, Ca, and P in the lentil microgreens. In addition, these Fabaceae microgreens may help in the nutritional security of the population residing in the high-altitude regions of Ladakh, especially during winter months when this region remains landlocked due to heavy snowfall.

## Introduction

Microgreens are 7 to 21-day-old, 3- to 8-cm-long seedlings mostly produced from the seeds of vegetables, herbs, and pulses and harvested at the first true leaf stage of plants (Xiao et al., 2012). Microgreens are gaining popularity during the last few years as they provide an array of textures, colors, flavors, and aromas to the food. They have outstanding nutritional and antioxidant properties and are also considered “functional foods” (Kyriacou et al., 2016). Microgreens are reported four to six times nutrient-rich than their mature counterparts (Xiao et al., 2012), as, during germination, there is an extensive breakdown of seed-storage compounds and simultaneous synthesis of structural proteins and other cell components (Danilcenko et al., 2017). Additionally, microgreens generate little or no food wastage during consumption as no biomass (except roots) gets wasted as trimming (Weber, 2017).

They differ from “sprouts” as they need light and a growing medium and have a longer growth cycle which may vary depending upon the species used, with an edible portion being stem and a pair of first true leaves (Xiao et al., 2012). Depending on the growth stage of the plant, the phytonutrient levels differ, and often a reduction is recorded from the seedling (sprout, microgreen) to the fully developed stage (Nakamura et al., 2001; Barillari et al., 2005).

In recent years, the microgreens market is growing rapidly (Charlebois, 2018; Riggio et al., 2019) and is also sold as a “living product” with the growing media. This helps consumers use them fresh as per their convenience (Renna et al., 2017). Till now, it has gained the market mostly in the western countries. However, in India, it is gaining popularity typically in the metro cities. The US is the major microgreens contributor in the year 2019 and was followed by Canada and Mexico. The global microgreens market is expected to grow at a CAGR of 7.5% from 2020 to 2025 (https://www.mordorintelligence.com/industry-reports/microgreens-market). Broccoli, lettuce, arugula, and basil are key microgreens grown across the regions under hydroponics and vertical farming.

Diet-related diseases like diabetes, obesity, hypertension, cancer, etc., are on the rise in both developing and developed countries, and are partly due to the imbalanced intake of the food and are mostly below recommended levels [World Health Organization and Food and Agriculture Organization of the United Nations (WHO/FAO), 2005; Choe et al., 2018]. Food supplementation through microgreens is known to modulate weight gain, cholesterol metabolism and protects against cardiovascular diseases (Pinto et al., 2015; Huang et al., 2016). Eradication of hidden global hunger and food insecurity is the key component of the sustainable development goals, and we should aim to achieve Zero Hunger and better nutrition by 2030 (www.fao.org; White and Broadley, 2009). For food diversification, microgreens are considered a novel product rich in several phytochemicals, including vitamins, antioxidants, and minerals. Increasing culinary demand and the ease of microgreens cultivation have generated a lot of interest in both growers and the consumers (Weber, 2017).

In South Asia, mungbean is consumed mainly as porridge/*dhal*, while in the rest of Asia, it is consumed as sprouts or noodles. Moreover, pulse sprouts have long been an essential, year-round component of Asian and vegan diets (Ebert et al., 2015a). Mungbean sprouts are used in different combination as a dietary supplement in healthcare; while lentil is also rich in fiber, protein, and complex carbohydrates (Gan et al., 2017). In addition, pulses are low in glycemic index, which makes them a good food choice for any diabetic person on a controlled diet (http://www.urbancultivator.net/herbguide/lentils/).

Rapid growth cycle, limited space requirement, and high economic produce make microgreens a nutrient alternative that may contribute to the nutritional security in the plain regions and the high-altitude areas of Ladakh (India) (Angmo et al., 2019). Ladakh is situated at 3,500 m AMSL and remains landlocked for 6 months due to heavy snowfall. Thus, this region needs novel and economically sustainable technology like microgreens (using cheap and abundant sources such as lentil and mungbean) in a big way to assure the nutritional security of the population residing in that region, especially during landlocked conditions of the winter months (Cohen and Garrett, 2010). Microgreens have the potential to contribute to food and nutritional security in the Ladakh sector as they can be easily grown at any altitude where land and low temperature is often a limiting factor, either under the greenhouse or even in the houses (near the glass window), independent of seasonal growth cycles (Ebert et al., 2015b, 2017). Light conditions are highly influential on the morpho-physiology of microgreens and the biosynthesis and accumulation of phytochemicals (Delian et al., 2015). However, there is scarce information about the nutritional properties of mungbean and lentil microgreens when grown under different environmental conditions. Against this backdrop, the present study has been carried out to find the comparative phytonutrient composition of a set of lentils and mungbean microgreens when grown under plain-altitude (Delhi) and high-altitude conditions of Leh-Ladakh (India).

## Materials and Methods

### Genotypes Used for Lentil and Mungbean Microgreens and Growing Conditions

**Figure 1:**
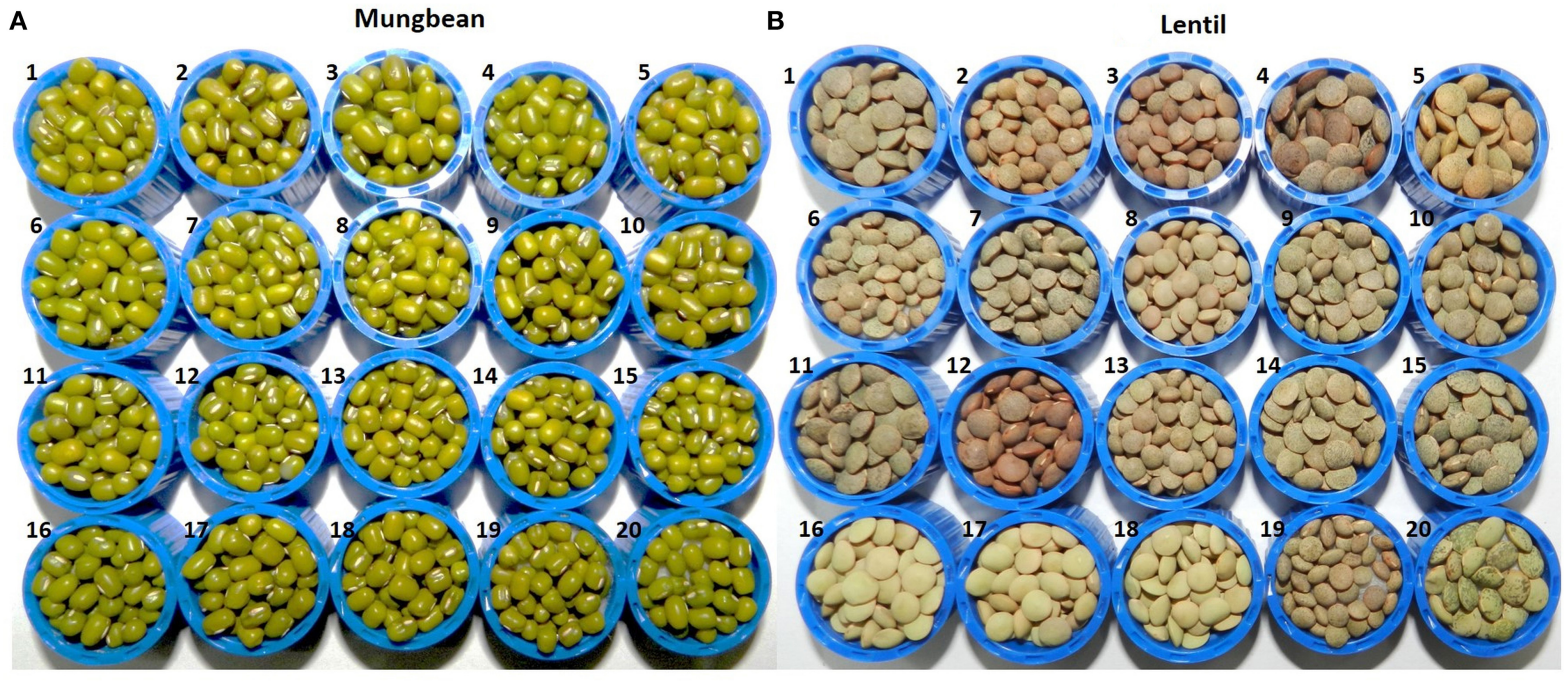
Seed morphology details of 20 genotypes each of **(A)** mungbean and **(B)** lentils used in the study, where **(A)** mungbean genotypes are (1) Pusa Baisakhi, (2) Pusa Ratna, (3) Pusa Vishal, (4) Pusa105, (5) Pusa0672, (6) Pusa9072, (7) Pusa9531, (8) MH96-1, (9) MH318, (10) MH421, (11) MH521, (12) MH810, (13) ML512, (14) ML818, (15) PS16, (16) TM 96-2, (17) IPM02-3, (18) IPM02-14, (19) IPM409-4, (20) PMR1; while **(B)** lentil genotypes are (1) L4076, (2) L4147, (3) L4594, (4) L7903, (5) HM1, (6) BM4, (7) JL1, (8) Sehore74-3, (9) NDL1, (10) IPL81, (11) IPL321, (12) K75, (13) KLS218, (14) DPL58, (15) DPL62, (16) PL1, (17) PL2, (18) PL6, (19) L830, (20) L4602.

To identify the genotypic differences for various biochemical parameters, a set of 20 diverse genotypes of mungbean and lentils were used for the analysis (Figure 1; Supplementary File 1). These were grown under partially controlled conditions in the National Phytotron Facility, IARI, New Delhi, located at the latitude and longitude of 28.6412°N, 77.1627°E, respectively, and an elevation of 228.61 m AMSL. The desired temperature was maintained for mungbean (28/26°C) and lentil (21/18°C) along with natural day and night cycles. Same genotypes were also grown under the greenhouse at Leh-Ladakh, which is situated at 3500 m AMSL at the latitude and longitude of 34.1383°N, 77.5727°E, respectively. The day length in terms of sunshine hours (h) was recorded as 10.4 ± 0.007 h and 10.3 ± 0.005 h at Leh and Delhi, respectively, during the microgreens growing period (Supplementary File 1).

The sample under study was sown during the first week of November 2019 at both Delhi and Leh conditions. Seeds were sown in three replicates in the seedling trays, having 50 cells per tray of cell-size 4.5 ×4.5 ×5.7 cm. The growing media consisted of coco peat: vermiculite: sand in the ratio of 2:1:1 for both mungbean and lentil. Harvesting of the microgreens was performed once it reached the optimum stage. Mungbean and lentil microgreens were harvested on the 7th and 9th days after sowing, respectively. Microgreens were manually collected using ethanol-cleaned scissors by cutting the stem ~1.0 cm above the growing medium. Harvested microgreens were immediately weighed using analytical balance to determine the total fresh weight (FW) and are used for various analyses. In addition, a set of microgreens were also dried in hot air GenLab vertical oven at 40.0°C for 72.0 h and are kept in airtight containers. Before analysis, the samples were kept in a vacuum desiccator for 24 h.

### Total Phenolics Content (TPC)

Total phenolics content was analyzed by modified Folin–Ciocalteu colorimetric method (Singleton et al., 1999) using regression equation of the calibration curve of Gallic acid (conc. from 50 to 500 μg/mL) and expressed as mg Gallic acid equivalents per g FW (mg GAE/g FW).

### Total Flavonoids Content (TFC)

Ethanolic extract was prepared by crushing the 0.1 g of sample in 80% ethanol, and to 0.5 mL sample, 0.5 mL of 2% AlCl3 ethanolic solution was added, which was then incubated for 1.0 h at room temperature, absorbance was measured at 420 nm. TFC was estimated as quercetin equivalent from a calibration curve (Woisky and Salatino, 1998).

### DPPH Scavenging Activity

The methanolic extract was prepared by crushing the 0.1 g sample in 1 mL of methanol, and this was used to determine the DPPH scavenging activity by measuring the absorbance of the mixture spectrophotometrically at 517 nm (Brand-Williams and Berset, 1995) and the ability to scavenge DPPH radical was calculated using the following equation:

(1) $$\begin{array}{r} \;DPPH\;scavenging\;activity\;\left(\%\right)\;=\\ \left[\left(Abs_{Control}-Abs_{Sample}\right)/\left(Abs_{Control}\right)\times 100\right] \end{array}$$

where Abs_Control_ is the absorbance of DPPH radical + methanol; Abs_Sample_ is the absorbance of DPPH radical + sample extract.

### FRAP Assay

For extract preparation, a 0.1 g sample was crushed using liquid N_2_, then 1.0 mL ethanol (80%) was added, and the homogenate was centrifuged (12,000 rpm; 15.0 min). The supernatant was collected, and 100 μL extract per genotype was used for FRAP reaction (Benzie and Strain, 1999). To this 3.0 mL pre-warmed freshly prepared FRAP reagent (acetate buffer: TPTZ: FeCl_3_.6H_2_O in 10:1:1 ratio) was added, and the mixture was incubated (37°C for 10.0 min) and increase in absorbance was measured using a spectrophotometer at 593 nm, and was compared with that of the standard calibration curve (20 mM FeSO_4_.7H_2_O), and the final concentration was expressed as mM/g of FW.

### Peroxide Quantification

Briefly, 0.1 g leaf samples were crushed in liquid N_2_, homogenized in 3.0 mL trichloroacetic acid (TCA; 1.0% w/v), centrifuged (10,000 rpm; 15 min at 4.0°C), and the supernatant was collected. Subsequently, 0.75 mL supernatant was mixed with 0.75 mL potassium phosphate buffer (10 mM; pH 7.0) and 1.5 mL freshly prepared potassium iodide (KI; 1.0 M) solution. The peroxide content was quantified in the supernatant by comparing the absorbance at 390 nm with that of the standard calibration curve ranging from 10 to 200 μmol/mL of H_2_O_2._ The final concentration was expressed as μmol/g of FW (Loreto and Velikova, 2001).

### Total Tocopherol Content (TTC)

Tocopherol content was determined by the bathophenanthroline method (Tsen, 1961), which utilizes iron (Fe) (III)-bathophenanthroline complex as the oxidizing agent. The sample extract was exposed to an ethanolic solution comprising 4,7-diphenyl-1,10-phenanthroline (Bathophenanthroline) and FeCl_3_, and to this H_3_PO_4_ was added after 15 s, and its stability was observed for 60 min. The absorbance of the solution was measured at 534 nm, and the tocopherol concentration was calculated from a standard curve and expressed in μg/g of FW.

### Total Carotenoids Content (TCC)

Briefly, 0.34 g of sample was crushed in liquid N_2_, then 6.0 mL ethyl alcohol:BHT (1.0 mg of BHT/mL of ethanol) was added and incubated at 85°C for 6 min with continuous vortexing. To this, 120 μL KOH was added and incubated for 5.0 min (at 85°C), then cooled on ice and then 4.0 mL distilled water and 3.0 mL PE:DE (2:1, v/v) was added. This was centrifuged for 10 min at room temperature, and the upper phase containing carotenoids was collected, and the solution was diluted to 10 mL (Lichtenthaler and Wellburn, 1983). The increase in absorbance was measured at 470 nm, and total carotenoids content was calculated using the formula:

(2) $$\begin{array}{r} \;Total\;carotenoid\;content\;\left(\mu g/g\right)\;=\\ \left[\left(A_{total}\;\times Vol\left(mL\right)\;\times \;\text{1000}\right)/\left(A^{1\text{\%}}\;\times Sampleweight\right)\right] \end{array}$$

where A_total_= absorbance at 450 nm; Vol (mL) = total volume of extract; A^1%^ = absorbance coefficient for carotenoid by column mixture.

### Ascorbic Acid

Ascorbic acid estimation was done through titration method using 2,6 dichloroindophenols dye solution and 4% oxalic acid as a stabilizing medium (Sadasivam and Balasubraminan, 1987; Nielsen, 2017). The dye solution was prepared in NaHCO_3_ hot solution, while dye standardization was done by titrating the standard ascorbic acid (1.0 mg/mL) to pale pink color, which persisted for 15.0 s, and calculation was done using the following formula:

(3) $$\begin{array}{r} \;Amount\;of\;Ascorbic\;acid\;\left(mg/100g\right)\;=\\ \left[\left(X_{mg}\;\times V_{2}\;\times Z_{mL}\right)/\left(V_{1}\;\times Y_{mL}\;\times Wt.\;of\;sample\right)\right]\;\times \;100 \end{array}$$

where X_mg_ = mg of standard ascorbic acid; V_1_ = Titer value of Standard ascorbic acid against dye; V_2_ = Titer value of sample against dye; Y_mL_ = Amount of aliquot taken (mL) for estimation; Z_mL_ = total amount (mL) of the extracted sample.

### Protein and Enzyme Extract Preparation

Briefly, 0.1 g fresh sample of each genotype was ground in liquid N_2_, then 3.0 mL of 0.1 M potassium phosphate buffer (pH 7.0) was added, and the homogenate was centrifuged at 13,000 rpm for 20 min at 4.0°C. The supernatant was used for the estimation of enzymatic activity and total soluble protein.

### Total Soluble Protein

The Bradford assay was used to determine the total soluble protein in the fresh lentil and mungbean samples (Bradford, 1976). The binding of protein molecules to Coomassie dye under acidic conditions results in a change in color from brown to blue, measured at 420 nm, and a calibration graph was prepared using 2, 4, 6, 8, 10 mg/mL BSA.

### Peroxidase (POD) Activity

For the estimation of POD activity (Lin and Kao, 1999), 976 μL potassium phosphate buffer (50.0 mM; pH 7.0) was mixed with 3.0 μL supernatant. To this, 10.0 μL guaiacol (900 mM) and 20.0 μL H_2_O_2_ (500 mM) were added, and guaiacol oxidation was determined by measuring the increase in absorbance at 470 nm. Enzyme activity was calculated using the extinction coefficient of 26.60 mM^−1^cm^−1^ for guaiacol and expressed as U min^−1^mg^−1^ protein of oxidized guaiacol.

### Catalase (CAT) Enzymatic Activity

CAT activity was estimated in the supernatant by measuring the decline in absorbance (decomposition of H_2_O_2_) at 240 nm (Aebi, 1984).

### Determination of Mineral Composition

#### Sample Preparation

The thoroughly dried samples were first homogenized using Agate mortar and pestle, and 100 mg powder was put in a cup having 23.9 mm aperture. The sample powder was gently pressed using an acrylic piston to prevent any void in the sample. The sample thickness was kept as 7.0 mm and was put in a vacuum desiccator for 24 h to make it completely moisture-free.

#### Sample Analysis Using Energy Dispersive X-ray Fluorescence Spectrometer (EDXRF)

Analyses of metals were carried out using EDXRF (Epsilon5 Spectrometer, PANalytical, United Kingdom) fitted with PAN-32 Ge detector. The 3-D polarized optics and low power improved the detection limit by lowering the spectral background for which samples were repeatedly used in the Epsilon 5 spectrometer. The resolution of the instrument was >150 eV. As the EDXRF scans the whole periodic table, the individual elements were detected at certain specific sites. During sampling, the instrument was run at a flow rate of 30.0 L/min for 24 h at all the sites. The EDXRF spectrometer was pre-calibrated before running, which has performed a semi-quantitative analysis of elements with 5–10% error. One blank was included for every 20 samples analyzed for quality assurance, and all the samples were run in triplets for accuracy.

### Photosynthetically Active Radiation (PAR), UV-B, and Photoperiod

The PAR and UV-B were recorded with a radiometer (PMA2100, Solar Light, USA), and the details of photoperiod were obtained from the Indian Agricultural Research Institute, New Delhi (India), and Defence Institute of High Altitude Research, Leh-Ladakh (India).

### Statistical Analysis

The experiments were conducted in three replications, and the results were presented as mean ± SD. One-way ANOVA was performed using SPSS11.5 to compare the groups, and Pearson's correlation test was used to assess the correlation between means. The mean comparison was performed using Tukey's test. Dendrograms were constructed using the Ward method, and distance is expressed as Euclid distance, and *P* <0.05 was regarded as significant. Principal component analysis (PCA) was performed on various antioxidant activities [e.g., DPPH, FRAP, peroxide, TTC, TCC, total ascorbic acid (TAA), POD, and CAT], protein content, phenolics [total phenolics content (TPC)], and flavonoids contents (TFC) in the mungbean and lentil microgreens when grown at normal altitude (Delhi) and high altitude (Leh) conditions. Analysis was done using STAR ver. 2.0.1 software and PCA plot were generated based on the first and second principal components (PC1 and PC2).

## Results and Discussion

### Total Phenolics Content (TPC)

The nutraceutical potential of microgreens is usually determined by their phenolics content which exhibits antioxidant, anti-inflammatory, and anticancer properties (Chon, 2013). TPC in the mungbean and lentil microgreens ranged from 190.92 to 240.81 and 212.60 to 276.98 mg GAE/100 g FW, respectively when grown at Delhi; while the same ranged from 200.34 to 241.75 and 218.23 to 321.35 mgGAE/100 g FW, respectively when grown under Leh conditions (Table 1). In mungbean, the genotype MH810 and in lentil the genotype DPL62 showed maximum TPC. Overall, TPC was found relatively more in the lentil microgreens (212.60 to 321.35 mgGAE/100 g FW) over mungbean microgreens (190.92–241.75 mgGAE/100 g FW). Similarly, a wide range of TPC ranging from 88.6 mgGAE/100 g FW in Upland cress to 811.2 mg GAE/100 g FW in Radish ruby has been reported (Xiao et al., 2019). However, no significant differences were recorded for either TPC or overall antioxidant capacity in broccoli microgreens when grown on either soil media or under hydroponic conditions (Tan et al., 2020).

**Table 1:** Mean concentration of total phenolics, flavonoids, carotenoids, tocopherols, and ascorbic acid in mungbean microgreens grown under Delhi and Leh conditions.

**S. No**.	**Genotype**	**TPC (mg GAE/100 g FW)**		**TFC (mg/100 g FW)**		**TTC (mg/100 g FW)**		**TCC (mg/100 g FW)**		**TAA (mg/100 g FW)**	
		**Delhi**	**Leh**	**Delhi**	**Leh**	**Delhi**	**Leh**	**Delhi**	**Leh**	**Delhi**	**Leh**
1.	Pusa Baisakhi	230.34 ± 5.78ab	234.09 ± 7.44abc	3.27 ± 0.20ab	4.41 ± 0.08fg	1.72 ± 0.058a	1.74 ± 0.176ab	7.03 ± 0.24ef	7.77 ± 0.33de	34.55 ± 1.28b	65.04 ± 2.99efg
2.	PusaRatna	234.67 ± 8.85ab	235.19 ± 13.26ab	2.92 ± 0.36b	5.12 ± 0.11ab	**1.73** **±** **0.035a**	1.68 ± 0.055abc	7.43 ± 0.47cdef	7.92 ± 0.21de	**32.37** **±** **1.45c**	63.53 ± 4.28efg
3.	Pusa Vishal	209.15 ± 5.21defg	203.63 ± 1.62de	3.44 ± 0.11ab	4.07 ± 0.09h	1.70 ± 0.057ab	**1.82** **±** **0.065a**	7.90 ± 0.33cde	8.72 ± 0.35cd	34.67 ± 1.45ab	73.81 ± 4.28bcd
4.	Pusa105	224.46 ± 5.21abcd	232.38 ± 9.58abc	3.29 ± 0.23ab	4.60 ± 0.17ef	1.59 ± 0.015cd	1.62 ± 0.102abcde	8.27 ± 0.28c	9.05 ± 0.35bc	34.30 ± 2.48bc	**88.63** **±** **2.99a**
5.	Pusa0672	**240.71** **±** **14.06a**	233.94 ± 16.20abc	2.93 ± 0.03b	4.57 ± 0.14ef	**1.17** **±** **0.027k**	**1.14** **±** **0.051g**	**6.98** **±** **0.26f**	7.37 ± 0.47e	33.94 ± 2.31bc	67.46 ± 7.27def
6.	Pusa9072	220.71 ± 5.21bcde	232.90 ± 7.37abc	2.99 ± 0.77b	4.25 ± 0.11gh	1.24 ± 0.009jk	1.28 ± 0.032fg	7.25 ± 0.59def	8.03 ± 0.19de	33.15 ± 1.37bc	76.23 ± 3.42bc
7.	Pusa9531	199.25 ± 4.17fg	207.90 ± 8.84de	**2.81** **±** **0.12b**	**4.06** **±** **0.10h**	1.29 ± 0.052ij	1.42 ± 0.079def	7.97 ± 0.14cd	8.27 ± 0.33cde	34.24 ± 2.22ab	82.58 ± 2.99ab
8.	MH96-1	194.88 ± 6.77fg	219.35 ± 1.47bcd	2.95 ± 0.29b	5.25 ± 0.07a	1.52 ± 0.045de	1.56 ± 0.048abcde	7.72 ± 0.31cdef	7.78 ± 0.54de	35.51 ± 1.11ab	71.69 ± 2.14cde
9.	MH318	202.69 ± 2.08efg	203.21 ± 2.21de	3.13 ± 0.25ab	5.13 ± 0.13ab	1.48 ± 0.017ef	1.51 ± 0.090bcdef	10.67 ± 0.38b	9.97 ± 0.14b	35.88 ± 1.45ab	76.84 ± 2.57bc
10.	MH421	207.06 ± 8.33defg	236.02 ± 10.31ab	2.95 ± 0.89b	5.17 ± 0.12a	1.47 ± 0.051ef	1.49 ± 0.044bcdef	11.43 ± 0.47ab	12.00 ± 0.38a	33.58 ± 1.28bc	65.04 ± 2.99efg
11.	MH521	196.96 ± 6.77fg	218.31 ± 8.84bcde	3.23 ± 0.14ab	4.73 ± 0.08cde	1.48 ± 0.085ef	1.42 ± 0.079bcdef	**12.22** **±** **0.59a**	12.07 ± 0.33a	34.36 ± 1.03bc	67.16 ± 4.28def
12.	**MH810**	***240.81±4.17a***	***241.75±2.21a***	***3.71±0.18a***	4.84 ± 0.15bcde	1.32 ± 0.034hij	1.40 ± 0.061def	***12.10±0.38a***	***12.10±0.80a***	***35.33±1.20ab***	***87.73±4.28a***
13.	ML512	194.25 ± 5.21fg	208.26 ± 4.94de	3.35 ± 0.23ab	4.64 ± 0.21def	1.62 ± 0.031bc	1.67 ± 0.163abcd	7.20 ± 0.47def	7.58 ± 0.59e	33.82 ± 0.94bc	65.46 ± 3.59efg
14.	ML818	**190.92** **±** **1.56g**	213.42 ± 10.46de	2.99 ± 0.22b	4.13 ± 0.12gh	1.70 ± 0.040ab	1.71 ± 0.103ab	7.27 ± 0.42def	7.67 ± 0.38e	34.55 ± 1.63b	65.34 ± 3.42efg
15.	PS16	192.27 ± 5.73fg	209.20 ± 1.10de	2.89 ± 0.11b	4.41 ± 0.33fg	1.59 ± 0.020cd	1.63 ± 0.052abcde	7.32 ± 0.54def	7.60 ± 0.66e	34.30 ± 1.11bc	**57.78** **±** **3.85g**
16.	TM96-2	192.79 ± 4.17fg	**200.34** **±** **5.52e**	3.09 ± 0.14ab	**5.22** **±** **0.14a**	1.57 ± 0.015cd	1.69 ± 0.056ab	7.12 ± 0.16def	**7.32** **±** **0.35e**	35.03 ± 1.97ab	63.83 ± 3.85efg
17.	IPM02-3	210.66 ± 5.36cdef	206.80 ± 7.29de	3.21 ± 0.12ab	5.07 ± 0.12ab	1.18 ± 0.027k	1.56 ± 0.371bcde	7.42 ± 0.31cdef	8.05 ± 0.35cde	**36.00** **±** **2.48a**	64.43 ± 2.99efg
18.	IPM02-14	219.56 ± 3.96bcde	216.75 ± 9.58cde	3.41 ± 0.15ab	4.95 ± 0.13abcd	1.44 ± 0.008efg	1.55 ± 0.047bcde	7.32 ± 0.49def	7.50 ± 0.61e	34.91 ± 1.11ab	61.11 ± 4.28fg
19.	IPM409-4	198.16 ± 5.78fg	220.45 ± 10.24bcd	3.37 ± 0.24ab	5.03 ± 0.14abc	1.36 ± 0.025ghi	1.43 ± 0.040cdef	7.28 ± 0.54def	7.43 ± 0.66e	34.73 ± 1.54ab	66.85 ± 2.14def
20.	PMR-1	229.56 ± 5.21abc	241.75 ± 5.16a	2.85 ± 0.29b	5.11 ± 0.08ab	1.40 ± 0.08gfh	1.41 ± 0.077ef	7.50 ± 0.24cdef	7.67 ± 0.57e	35.21 ± 2.40ab	62.62 ± 2.99fg

*TPC, total phenolics content; TFC, total flavonoids content; TCC, total carotenoids content; TTC, total tocopherols content; TAA, total ascorbic acid. Values are expressed as mean ± SD (n = 3), and different letters indicate a significant difference (p ≤ 0.05)*.

### Total Flavonoids Content (TFC)

Flavonoids generally have more antioxidant activity (AoA) than phenolics (Heim et al., 2002), and they have anticancer properties due to their role in the signal transduction pathways of cell division (Aron and Kennedy, 2008). When grown at Leh, TFC was found significantly more for mungbean microgreens (4.06–5.25 mg/100 g FW) than the lentil microgreens (1.06–2.84 mg/100 g FW) and was also higher than those of the Delhi-grown mungbean (2.81–3.71 mg/100 g FW) and lentil (0.98–1.86 mg/100 g FW) microgreens (Tables 1, 2). Similarly, the flavonoids in other culinary microgreens like mustard (1.1 mg/100 g FW) and roselle (6.5 mg/100 g FW) were recorded in a similar range (Ghoora et al., 2020). In addition, Swieca and Gwalik-Dziki (2015) recorded TFC in the 6-day sprouts of mungbean (0.57 mg/g DW) and lentil (0.57 mg/g DW); whereas, Ebert et al. (2017) reported flavonoids (as Kaempferol) in the sprouts of different mungbean genotypes in the range of 0.32 to 0.40 μmol/100 g.

**Table 2:** Mean concentration of total phenolics, flavonoids, carotenoids, tocopherols, and ascorbic acid in lentil microgreens grown under Delhi and Leh conditions.

**S. No**.	**Genotype**	**TPC (mg GAE/100 g FW)**		**TFC (mg/100 g FW)**		**TTC (mg/100 g FW)**		**TCC (mg/100 g FW)**		**TAA (mg/100 g FW)**	
		**Delhi**	**Leh**	**Delhi**	**Leh**	**Delhi**	**Leh**	**Delhi**	**Leh**	**Delhi**	**Leh**
1	L4076	259.35 ± 12.20abcde	290.10 ± 6.19bcde	1.66 ± 0.023c	2.14 ± 0.023d	1.10 ± 0.014e	1.11 ± 0.006efgh	**14.18** **±** **0.08h**	14.70 ± 0.368i	16.94 ± 0.86fghi	24.50 ± 0.43efg
2	L4147	235.85 ± 8.66efghi	245.73 ± 7.07hij	1.23 ± 0.068g	1.39 ± 0.068f	1.01 ± 0.070g	1.10 ± 0.024fgh	14.48 ± 0.17gh	16.10 ± 0.198h	17.12 ± 0.26fghi	25.53 ± 0.17cde
3	L4594	260.73 ± 14.14abcd	302.60 ± 6.19abc	1.83 ± 0.034a	2.11 ± 0.091d	1.08 ± 0.004ef	1.20 ± 0.232defg	20.4 ± 0.06a	20.70 ± 0.085bc	17.91 ± 0.51ef	26.74 ± 0.17ab
4	L7903	248.23 ± 8.84bcdefg	276.98 ± 10.61def	1.72 ± 0.011bc	2.14 ± 0.113d	1.07 ± 0.007efg	1.05 ± 0.007h	18.52 ± 0.23b	18.56 ± 0.113e	17.00 ± 0.94fghi	24.64 ± 0.60efg
5	HM1	222.54 ± 5.04hi	228.85 ± 16.79ijk	**1.86** **±** **0.034a**	2.58 ± 0.079b	1.24 ± 0.013ab	1.09 ± 0.014gh	16.82 ± 0.48cd	17.02 ± 0.198g	**16.15** **±** **0.26i**	25.17 ± 0.34cdef
6	BM4	266.35 ± 4.42ab	288.23 ± 5.30bcde	**0.98** **±** **0.102h**	1.22 ± 0.011gh	1.12 ± 0.014cde	1.12 ± 0.008efgh	17.16 ± 0.17c	17.82 ± 0.311f	16.64 ± 0.43hi	25.71 ± 0.43bcd
7	JL1	241.98 ± 3.54cdefgh	292.60 ± 4.42bcd	1.23 ± 0.045g	1.39 ± 0.045f	1.22 ± 0.029ab	1.21 ± 0.021cdefg	15.34 ± 0.82fg	**14.40** **±** **0.453i**	16.76 ± 0.26ghi	25.77 ± 0.51bc
8	Sehore74-3	218.48 ± 18.56hi	224.48 ± 12.37jk	1.39 ± 0.023f	1.94 ± 0.068e	1.02 ± 0.025fg	1.11 ± 0.022efgh	15.5 ± 0.08ef	15.76 ± 0.283h	17.48 ± 0.09fgh	24.50 ± 0.43efg
9	NDL-1	252.23 ± 11.49bcdef	281.35 ± 7.95cde	1.54 ± 0.091d	2.66 ± 0.091b	1.20 ± 0.023ab	1.22 ± 0.024cdefg	14.74 ± 0.54fgh	15.68 ± 0.280h	17.30 ± 0.51fgh	24.68 ± 1.03defg
10	IPL81	**212.60** **±** **6.19i**	225.73 ± 17.68jk	1.42 ± 0.045ef	2.32 ± 0.181c	**1.25** **±** **0.008a**	1.24 ± 0.018cde	16.44 ± 0.34cd	16.68 ± 0.283g	17.18 ± 0.34fghi	25.53 ± 0.17cde
11	IPL321	221.35 ± 11.49hi	224.48 ± 1.77jk	1.53 ± 0.057de	2.61 ± 0.113b	1.22 ± 0.019ab	1.23 ± 0.005cdef	16.66 ± 0.48cd	20.48 ± 0.170bc	17.79 ± 0.34efg	26.02 ± 0.17abc
12	K75	251.98 ± 11.25bcdef	270.73 ± 15.91efg	1.82 ± 0.045ab	2.62 ± 0.045b	1.21 ± 0.007ab	**1.40** **±** **0.010a**	20.58 ± 0.20a	20.96 ± 0.171b	17.91 ± 0.34ef	**26.86** **±** **0.34a**
13	KLS218	271.98 ± 15.91ab	307.60 ± 9.72ab	1.22 ± 0.034g	**1.06** **±** **0.034h**	1.12 ± 0.004cde	1.34 ± 0.132abc	19.14 ± 0.59b	19.22 ± 0.141d	**21.42** **±** **0.51a**	24.26 ± 0.09fg
14	DPL58	261.91 ± 5.21abc	287.60 ± 6.19bcde	1.24 ± 0.011g	1.24 ± 0.011fg	1.21 ± 0.036ab	1.24 ± 0.013cde	19.32 ± 0.34b	19.4 ± 0.226d	21.30 ± 0.68a	23.96 ± 0.34g
15	DPL62	**276.98** **±** **15.91a**	**321.35** **±** **7.95a**	1.25 ± 0.045g	1.23 ± 0.068fg	1.10 ± 0.024de	1.19 ± 0.030defg	19.36 ± 0.57b	20.32 ± 0.226c	18.57 ± 0.60de	**23.84** **±** **0.51g**
16	PL1	234.48 ± 8.84fghi	**218.23** **±** **5.30k**	1.23 ± 0.068g	1.13 ± 0.011gh	1.21 ± 0.005ab	1.26 ± 0.010bcd	17.1 ± 0.54cd	17.98 ± 0.424f	19.84 ± 0.17bc	24.02 ± 0.60g
17	PL2	224.16 ± 10.16ghi	235.73 ± 8.84hijk	1.62 ± 0.011cd	2.50 ± 0.091b	1.18 ± 0.034bc	1.16 ± 0.059defgh	16.24 ± 0.28de	16.66 ± 0.255g	19.66 ± 0.43c	24.02 ± 0.0.60g
18	PL6	231.29 ± 7.87fghi	250.10 ± 7.95ghi	1.23 ± 0.068g	2.23 ± 0.011cd	1.17 ± 0.036bcd	1.17 ± 0.018defgh	16.76 ± 0.34cd	18.08 ± 0.170ef	19.00 ± 0.34cd	24.50 ± 0.43efg
19	**L830**	234.23 ± 10.78fghi	255.85 ± 12.20fgh	***1.86±0.023a***	***2.84±0.057a***	***1.21±0.005ab***	***1.38±0.019ab***	***20.62±0.20a***	***22.22±0.198a***	***20.75±0.26ab***	25.71 ± 0.43bcd
20	L4602	236.85 ± 8.84defgh	285.10 ± 9.72cde	1.37 ± 0.034f	2.09 ± 0.079de	**1.00** **±** **0.089g**	**1.04** **±** **0.009h**	17.02 ± 0.37cd	18.08 ± 0.057ef	18.57 ± 0.60de	26.14 ± 0.68abc

*TPC, total phenolics content; TFC, total flavonoids content; TCC, total carotenoids content; TTC, total tocopherols content; TAA, total ascorbic acid. Values are expressed as mean ± SD (n = 3), and different letters indicate a significant difference (p ≤ 0.05)*.

### Total Tocopherol Content (TTC)

Among lipophilic antioxidants, the TTC in the mungbean and lentil microgreens were recorded ranging from 1.17–1.73 and 1.0–1.25 mg/100 g FW, respectively, when grown at Delhi; while the same ranged from 1.14–1.82 and 1.04–1.4 mg/100 g FW when grown under Leh conditions (Tables 1, 2). Overall, the mungbean (Pusa Ratna and Pusa Vishal) and lentil (L830 and K75) genotypes expressed higher TTC (Tables 1, 2). However, relatively higher TTC was recorded in brassica-based microgreens, ranging from 1.8 mg/100 g FW in watercress to 5.1 mg/100 g FW in red radish (Xiao et al., 2019).

### Total Carotenoids Content (TCC)

The antioxidant capacity of the carotenoid is due to the presence of conjugated double bonds, which gives them the free radical scavenging property (Yang et al., 2018). When grown at Leh, the TCC was found significantly less for mungbean microgreens (7.32–12.10 mg/100 g FW) than the lentil microgreens (14.40–22.22 mg/100 g FW) but was higher than those of the Delhi-grown mungbean (6.98–12.22 mg/100 g FW) and lentil (14.18–20.62 mg/100 g FW) microgreens (Tables 1, 2). The mungbean genotypes, MH521 and MH810, and lentil genotypes L830 and L4594 were better for TCC. Nearly similar TCC was also recorded among 30 brassica microgreens which ranged from 8.2 (Pak Choy) to 18.8 mg/100 g FW (Upland cress) (Xiao et al., 2019). However, Brazaityte et al. (2015b) recorded relatively more TCC in mustard (17.06), red Pak Choi (40.43), and tatsoi (36.03 mg 100/g FM) microgreens under normal illumination.

### Total Ascorbic Acid (TAA)

The TAA in mungbean and lentil microgreens was recorded from 32.37 to 36.00 and 16.15 to 21.42 mg/100 g FW, respectively, when grown in Delhi, and the same ranged from 61.11 to 88.63 and 23.84 to 26.86 mg/100 g FW, respectively, when grown under Leh conditions. Significantly more TCC were recorded for the mungbean genotypes IPM02-3, MH310, and Pusa105, while lentil genotypes L830, KLS218, and K78 showed relatively higher TAA content (Tables 1, 2). Similarly, in various brassica-derived microgreens like pepper cress and cauliflower, the TAA was recorded to the tune of 32.9 and 120.8 mg/100 g FW, respectively (Xiao et al., 2019). Similarly, significantly higher vitamin C content was recorded for the soil-grown broccoli microgreens over hydroponically grown samples (Tan et al., 2020).

### Non-enzymatic AoA (DPPH, FRAP, and H_2_O_2_ Assay)

Since different mechanisms are associated with the AoA of any plant; thus, this can be determined through various ways capturing varied modes of action like reducing abilities (FRAP), antiradical ability (DPPH), preventive effect against lipid (H_2_O_2_), and ability to chelate transition metals ions (Prior et al., 2005). Significantly more total AoA was recorded when mungbean microgreens were grown at Leh (FRAP: 11.73–20.22 μmol TE/g FW; DPPH: 3.2–3.79 μmol TE/g FW; H_2_O_2_: 5.08–6.14 nmol/g FW) than when grown at Delhi (FRAP: 5.7–11.30 μmol TE/g FW; DPPH: 0.69–1.585 μmol TE/g FW; H_2_O_2_: 2.61–4.74 nmol/g FW). In lentil too, the total AoA have recorded more for the Leh-grown microgreens (FRAP: 21.09–55.00 μmol TE/g FW; DPPH: 2.87–3.38 μmol TE/g FW; H_2_O_2_: 4.55–5.92 nmol/g FW) than the Delhi-grown samples (FRAP: 14.7–37.94 μmol TE/g FW; DPPH: 0.84–1.55 μmol TE/g FW; H_2_O_2_: 1.63–2.24 nmol/g FW). Overall, the mungbean genotype MH810 and lentil genotype L830 recorded higher total non-enzymatic AoA (Tables 3, 4).

**Table 3:** Total antioxidant activity, crude protein, and antioxidant enzymatic activities in the mungbean microgreens grown under Delhi and Leh conditions.

**S. No**.	**Genotypes**	**FRAP (μmol TE/g FW)**		**DPPH (μmol TE/g FW)**		**H**_****2****_ **O**_****2****_ **(nmol/g FW)**		**Crude protein (g/100 g FW)**		**Peroxidase Activity (U/g FW)**		**Catalase Activity (U/g FW)**	
		**Delhi**	**Leh**	**Delhi**	**Leh**	**Delhi**	**Leh**	**Delhi**	**Leh**	**Delhi**	**Leh**	**Delhi**	**Leh**
1	Pusa Baisakhi	9.91 ± 0.515abc	18.44 ± 0.980abcd	1.139 ± 0.131bcd	3.33 ± 0.039cd	3.75 ± 0.059fg	5.54 ± 0.124abcdegh	3.40 ± 0.01e	2.35 ± 0.04gh	292.71 ± 3.51bcd	**307.37** **±** **5.32e**	383.76 ± 43.07bcdefgh	456.85 ± 43.07abc
2	Pusa Ratna	9.90 ± 0.707abc	19.29 ± 1.263abc	1.176 ± 0.105bc	3.55 ± 0.380abc	2.91 ± 0.041l	5.47 ± 0.041abcdefg	3.35 ± 0.06ef	2.83 ± 0.08a	329.77 ± 50.40abc	458.01 ± 31.74abcd	398.98 ± 25.84abcdef	347.21 ± 8.61ghi
3	Pusa Vishal	9.63 ± 0.874bc	16.15 ± 0.990efg	1.069 ± 0.111bcde	**3.79** **±** **0.334a**	3.13 ± 0.024jk	5.31 ± 0.065defg	3.58 ± 0.24abcde	2.46 ± 0.14fgh	336.92 ± 19.25ab	452.14 ± 39.40bcd	370.05 ± 28.00bcdefgh	345.69 ± 32.30ghi
4	Pusa105	9.49 ± 0.510bc	18.35 ± 0.505bcd	1.116 ± 0.137bcd	3.36 ± 0.144cd	3.22 ± 0.059ij	5.86 ± 0.253abcde	3.39 ± 0.04e	2.56 ± 0.11cdefgh	302.33 ± 10.10bcd	445.60 ± 34.29cd	321.32 ± 10.77h	350.25 ± 17.23gh
5	Pusa0672	10.44 ± 2.015ab	20.25 ± 0.970ab	1.315 ± 0.144ab	3.32 ± 0.065cd	3.41 ± 0.058hi	6.09 ± 0.194ab	3.45 ± 0.01de	2.78 ± 0.10abc	281.95 ± 52.10cd	427.33 ± 17.39cd	421.83 ± 19.38abc	**470.56** **±** **23.69a**
6	Pusa9072	9.46 ± 0.495bcd	17.31 ± 1.035cdef	1.102 ± 0.132bcd	3.49 ± 0.033bcd	2.92 ± 0.041kl	5.90 ± 0.919abcd	3.57 ± 0.01abcde	2.81 ± 0.07ab	312.93 ± 11.06abcd	460.83 ± 49.44abcd	397.46 ± 23.69abcdef	391.37 ± 36.61bcdefgh
7	Pusa9531	8.63 ± 0.450cde	15.03 ± 0.490gh	1.046 ± 0.105bcdef	3.44 ± 0.052bcd	**2.61** **±** **0.024m**	5.56 ± 0.613abcdefg	3.52 ± 0.05abcde	2.53 ± 0.12defgh	**355.41** **±** **49.23a**	466.58 ± 20.26abcd	423.35 ± 21.54abcde	371.57 ± 38.77defgh
8	MH96-1	7.74 ± 0.495e	13.88 ± 0.909hi	0.889 ± 0.039cdefg	3.33 ± 0.039cd	3.45 ± 0.018h	5.82 ± 0.206abcdef	3.52 ± 0.34abcde	**2.87** **±** **0.15a**	325.04 ± 5.42abc	446.28 ± 8.77cd	426.40 ± 43.07abcd	360.91 ± 40.92fgh
9	MH318	8.79 ± 0.455cde	14.94 ± 1.364gh	1.051 ± 0.098bcdef	3.69 ± 0.046ab	3.44 ± 0.006h	5.33 ± 0.324cdefg	3.51 ± 0.15abcde	2.37 ± 0.06gh	325.19 ± 7.12abc	473.01 ± 34.77abcd	342.64 ± 28.00fgh	434.01 ± 49.53abcde
10	MH421	9.40 ± 0.303bcd	16.04 ± 1.010efg	1.065 ± 0.105bcde	3.72 ± 0.105ab	4.74 ± 0.065b	6.04 ± 0.583abc	3.70 ± 0.02ab	2.72 ± 0.16abcde	307.52 ± 5.32abcd	474.47 ± 21.21abcd	383.76 ± 64.61bcdefgh	441.62 ± 68.92abcd
11	MH521	8.00 ± 0.343de	15.32 ± 0.919fgh	0.949 ± 0.007cdefg	**3.20** **±** **0.144d**	4.60 ± 0.035bc	5.95 ± 0.483abcd	3.38 ± 0.08ef	2.69 ± 0.12abcdef	338.12 ± 30.09ab	458.68 ± 39.71abcd	395.94 ± 30.15abcdefgh	362.44 ± 30.15efgh
12	**MH810**	***11.30±0.510a***	***20.44±1.100a***	***1.585±0.421a***	***3.55±0.111abc***	3.91 ± 0.171f	***6.14±0.065a***	***3.75±0.02a***	***2.74±0.15abcde***	***326.92±10.63abc***	***513.50±33.65ab***	***467.51±36.61a***	***462.94±12.92ab***
13	ML512	7.81 ± 0.596e	13.81 ± 1.010hi	0.843 ± 0.065defg	3.52 ± 0.039abc	3.57 ± 0.301gh	**5.08** **±** **0.088g**	3.45 ± 0.01cde	2.58 ± 0.05bcdefg	321.20 ± 5.53abc	482.03 ± 7.97abc	322.84 ± 55.99gh	429.44 ± 17.23abcdef
14	ML818	**5.70** **±** **0.500f**	**11.73** **±** **0.465j**	**0.690** **±** **0.020g**	3.56 ± 0.052abc	2.96 ± 0.200kl	5.35 ± 0.047cdef	3.42 ± 0.02e	2.34 ± 0.13h	322.48 ± 12.65abc	452.93 ± 8.29bcd	**239.09** **±** **10.79i**	368.53 ± 25.84efgh
15	PS16	5.72 ± 0.551f	12.40 ± 0.101ij	0.759 ± 0.079fg	3.58 ± 0.033abc	**5.14** **±** **0.053a**	5.13 ± 0.035fg	3.38 ± 0.04e	2.51 ± 0.06efgh	317.29 ± 14.89abcd	419.21 ± 38.76d	351.78 ± 40.92defgh	**275.63** **±** **58.15i**
16	TM96-2	6.07 ± 0.460f	12.65 ± 0.247ij	0.796 ± 0.118efg	3.60 ± 0.013abc	3.76 ± 0.483fg	5.39 ± 0.053bcdefg	**3.15** **±** **0.28f**	2.45 ± 0.08gh	325.56 ± 12.76abc	**518.80** **±** **33.49a**	365.48 ± 47.38cdefgh	385.28 ± 6.46cdefgh
17	IPM02-3	9.40 ± 0.455bcd	16.74 ± 0.379defg	1.074 ± 0. 118bcde	3.48 ± 0.046bcd	2.78 ± 0.012lm	5.36 ± 0.106cdefg	3.68 ± 0.08abc	**2.34** **±** **0.05h**	268.35 ± 5.42d	457.22 ± 28.39abcd	441.62 ± 21.54ab	405.08 ± 21.54abcdefg
18	IPM02-14	9.39 ± 0.505bcd	16.65 ± 0.854defg	1.088 ± 0.0.111bcde	3.50 ± 0.072abc	4.18 ± 0.053e	5.18 ± 0.171efg	3.44 ± 0.04cde	2.75 ± 0.15abcd	**266.17** **±** **9.57d**	511.80 ±± 15.95ab	**465.99** **±** **43.07a**	367.01 ± 10.77efgh
19	IPM409-4	7.84 ± 0.434e	13.82 ± 1.212hi	1.019 ± 0.092bcdef	3.51 ± 0.092abc	4.37 ± 0.041de	5.15 ± 0.029efg	3.47 ± 0.02bcde	2.54 ± 0.13defgh	321.65 ± 25.52abc	459.70 ± 6.70abcd	420.30 ± 8.61abcde	446.19 ± 19.38abc
20	PMR-1	9.51 ± 0.505bc	17.87 ± 1.515cde	1.125 ± 0.124bcd	3.44 ± 0.105bcd	4.41 ± 0.047cd	6.10 ± 0.183ab	3.67 ± 0.01abcd	2.55 ± 0.04cdefgh	304.96 ± 4.04abccd	476.84 ± 31.26abcd	347.21 ± 25.84efgh	319.80 ± 17.23hi

*Values are expressed as mean ± SD (n = 3), and different letters indicate a significant difference (p ≤ 0.05)*.

Similarly, a wide range of DPPH activity was recorded in the upland cress (1.57 μmol TE/g FW) and radish ruby-based microgreens (8.06 μmol TE/g FW) (Xiao et al., 2019). However, relatively higher DPPH activity was also recorded in basil (9.60 μmol/g), beet (7.91 μmol/g), and pak choi (7.65 μmol/g) (Brazaityte et al., 2015a). Additionally, the mustard microgreens produced from cold plasma (at 21 and 23 kV) treated seeds showed significantly more DPPH activity over control (Saengha et al., 2021). FRAP activity in onion microgreens was recorded as 7.0 μmol Fe^2+^/g, while this was 36.3 μmol Fe^2+^/g in roselle (Ghoora et al., 2020). H_2_O_2_ in leaf tissue is known to respond to environmental stimuli (Cheeseman, 2006). Hydrogen peroxide acts as a signaling molecule in the plant system and imparts a second line of defense, and its increase is associated with oxidative damage (Ozden et al., 2009). There are only very few reports on peroxide content estimation in microgreens. In *Bruguiera parviflora* leaf tissue, the concentrations of H_2_O_2_ were reported increasing from 0.067 to 0.089 μmol (gFW)^−1^ when salinization treatment was given (Parida and Das, 2005). A similar increasing trend of H_2_O_2_ content was also recorded for Leh-grown microgreens over Delhi-grown microgreens.

### Total Crude (Soluble) Protein Content

Microgreens showed large variations for total crude protein content between genotypes and also between mungbean and lentil species. When grown in Delhi, the mungbean microgreens showed more crude protein content (3.15–3.75 mg/100 g FW) than when grown at Leh conditions (2.34–2.87 mg/100 g FW). Also, in the lentil microgreens, the protein content was recorded more for the Delhi samples (2.18–2.67 mg/100 g FW) over Leh-grown samples (1.84–2.16 mg/100 g FW). The lentil genotype L830 and mungbean genotype MH810 showed maximum protein content (Tables 3, 4). Similarly, in *Cichorium intybus* and *Brassica oleracea* derived microgreens, the protein contents were estimated as 1.9 and 3.0 mg/100 FW, respectively (Paradiso et al., 2018). More protein content was recorded for the mungbean microgreens among lentil and mungbean microgreens when grown at either Delhi or Leh conditions.

**Table 4:** Total antioxidant activity, crude protein, and enzymatic antioxidant activities of lentil microgreens grown under Delhi and Leh environmental conditions.

**S. No**.	**Genotypes**	**FRAP (μmol TE/g FW)**		**DPPH (μmol TE/g FW)**		**H**_****2****_ **O**_****2****_ **(nmol/g FW)**		**Protein (g/100 g FW)**		**Peroxidase Activity (U/g FW)**		**Catalase Activity (U/g FW)**	
		**Delhi**	**Leh**	**Delhi**	**Leh**	**Delhi**	**Leh**	**Delhi**	**Leh**	**Delhi**	**Leh**	**Delhi**	**Leh**
1	L4076	25.24 ± 1.49de	36.36 ± 0.13efg	1.08 ± 0.013c	3.15 ± 0.026cde	2.347 ± 0.059ab	4.551 ± 0.018b	2.22 ± 0.02de	1.91 ± 0.04bc	341.97 ± 6.93fg	466.41 ± 17.23abc	193.40 ± 2.15efg	370.05 ± 49.53abcde
2	L4147	30.76 ± 0.37b	44.61 ± 3.21bc	0.92 ± 0.039c	3.06 ± 0.065efg	2.138 ± 0.177abc	5.176 ± 0.041ab	2.28 ± 0.17cde	1.99 ± 0.13abc	437.59 ± 19.57a	478.52 ± 67.64ab	**159.90** **±** **36.61g**	367.01 ± 88.30abcde
3	L4594	19.34 ± 0.30gh	27.88 ± 1.21ij	1.19 ± 0.065bc	3.14 ± 0.020cde	1.959 ± 0.242bcdef	4.784 ± 0.194b	2.56 ± 0.13abcd	1.88 ± 0.02bc	418.94 ± 4.36ab	495.10 ± 16.64a	190.36 ± 40.92efg	261.93 ± 17.23ghi
4	L7903	18.11 ± 1.60h	26.03 ± 0.80j	1.09 ± 0.020c	3.17 ± 0.026cd	2.355 ± 0.024ab	**4.601** **±** **0.289b**	2.24 ± 0.06cde	2.04 ± 0.22abc	369.67 ± 13.97defg	459.18 ± 57.78abc	292.39 ± 30.15abc	333.50 ± 62.46bcdefg
5	HM1	**14.70** **±** **0.42i**	**21.09** **±** **0.62k**	**1.55** **±** **0.483a**	3.21 ± 0.039c	1.780 ± 0.012cdef	4.872 ± 0.059b	**2.18** **±** **0.02e**	2.01 ± 0.06abc	380.14 ± 40.84bcdef	491.87 ± 20.44a	216.24 ± 4.31defg	287.82 ± 58.15efghi
6	BM4	23.26 ± 0.36ef	33.61 ± 1.48gh	**0.84** **±** **0.039c**	3.00 ± 0.052g	1.705 ± 0.035def	4.713 ± 0.047b	2.34 ± 0.06abcde	1.91 ± 0.08bc	394.48 ± 16.97abcde	437.63 ± 10.54abc	289.34 ± 8.61abc	321.32 ± 23.69bcdefgh
7	JL1	24.68 ± 0.12de	35.69 ± 1.95efg	1.00 ± 0.046c	3.04 ± 0.072fg	1.659 ± 0.041ef	4.772 ± 0.200b	2.39 ± 0.16abcde	1.98 ± 0.07abc	414.62 ± 10.75abc	**401.53** **±** **10.49c**	289.34 ± 21.54abc	300.00 ± 32.30defghi
8	Sehore74-3	27.06 ± 1.57cd	39.11 ± 0.04de	1.05 ± 0.033c	3.11 ± 0.026def	**1.638** **±** **0.035f**	4.988 ± 0.189ab	2.40 ± 0.12abcde	1.96 ± 0.13abc	427.32 ± 4.07a	437.53 ± 46.88abc	309.14 ± 19.38ab	389.85 ± 47.38abcd
9	NDL-1	19.42 ± 1.68gh	27.93 ± 0.80ij	1.08 ± 0.007c	**3.38** **±** **0.013a**	1.672 ± 0.047ef	4.808 ± 0.487b	2.34 ± 0.17abcde	1.93 ± 0.14bc	346.87 ± 17.62fg	460.29 ± 36.80abc	313.71 ± 43.07ab	402.03 ± 21.54ab
10	IPL-81	23.36 ± 1.41ef	33.70 ± 0.05fgh	1.06 ± 0.026c	3.18 ± 0.007cd	**2.384** **±** **0.065a**	4.904 ± 0.308b	2.29 ± 0.02bcde	1.88 ± 0.07bc	**335.33** **±** **10.30g**	410.47 ± 9.13bc	190.36 ± 28.00efg	347.21 ± 21.54abcdef
11	IPL321	26.81 ± 0.04cd	38.81 ± 2.25de	1.06 ± 0.026c	3.31 ± 0.020ab	2.309 ± 0.065ab	5.279 ± 0.579ab	2.25 ± 0.14cde	1.87 ± 0.02c	346.37 ± 11.04fg	449.87 ± 78.51abc	257.36 ± 36.61abcd	274.11 ± 12.92fghi
12	K75	**37.94** **±** **2.04a**	**55.00** **±** **0.33a**	1.11 ± 0.013c	3.34 ± 0.046a	2.243 ± 0.147ab	4.902 ± 0.676b	2.33 ± 0.07abcde	**1.84** **±** **0.04c**	380.71 ± 17.36bcdef	485.73 ± 17.71ab	251.27 ± 19.38bcde	365.48 ± 30.15abcde
13	KLS218	30.26 ± 0.95b	43.90 ± 4.01bc	0.92 ± 0.007c	**2.87** **±** **0.065h**	2.251 ± 0.053ab	5.435 ± 0.335ab	2.58 ± 0.36abc	1.94 ± 0.11abc	406.95 ± 2.26abcd	479.59 ± 18.48ab	184.26 ± 40.92fg	237.56 ± 38.77hi
14	DPL58	21.44 ± 0.47fg	30.94 ± 1.16hi	1.01 ± 0.059c	3.03 ± 0.052fg	2.026 ± 0.572abcdef	5.239 ± 0.494ab	2.24 ± 0.27cde	2.09 ± 0.08ab	401.83 ± 22.09abcd	**502.80** **±** **24.64a**	251.27 ± 2.15bcde	345.69 ± 75.38abcdefh
15	DPL62	26.12 ± 0.54cde	37.78 ± 1.46de	1.02 ± 0.052c	2.99 ± 0.013g	2.122 ± 0.059abcd	4.813 ± 0.648b	2.55 ± 0.34abcd	1.93 ± 0.09bc	367.11 ± 28.68defg	436.77 ± 32.57abc	**319.80** **±** **38.77a**	**217.77** **±** **2.15i**
16	PL1	25.66 ± 0.23cde	37.12 ± 1.88efg	0.96 ± 0.013c	2.98 ± 0.092g	2.063 ± 0.519abcde	5.128 ± 0.356ab	2.22 ± 0.13de	2.03 ± 0.12abc	371.76 ± 10.74cdefg	496.95 ± 31.90a	239.09 ± 15.08cdef	363.96 ± 2.15abcdef
17	PL2	28.53 ± 3.20bc	41.18 ± 2.20cd	1.03 ± 0.072c	3.23 ± 0.059bc	2.138 ± 0.047abc	5.075 ± 0.820ab	2.55 ± 0.08abcd	1.88 ± 0.06bc	**428.89** **±** **22.38a**	426.66 ± 19.99abc	280.20 ± 34.46abc	335.03 ± 43.07bcdefg
18	PL6	25.95 ± 2.46cde	37.45 ± 1.35def	0.97 ± 0.013c	3.14 ± 0.020cde	2.180 ± 0.035abc	**5.925** **±** **0.735a**	2.57 ± 0.09abcd	1.92 ± 0.05bc	348.19 ± 26.80fg	472.14 ± 23.63abc	278.68 ± 40.92abcd	391.37 ± 10.77abc
19	**L830**	31.28 ± 0.73b	45.31 ± 1.65b	***1.53±0.509ab***	***3.37±0.033a***	***2.247±0.047ab***	4.684 ± 0.666b	***2.67±0.16a***	***2.16±0.15a***	***395.51±35.79abcd***	***495.84±22.94a***	237.56 ± 25.84cdef	***435.53±38.77a***
20	L4602	26.00 ± 0.33cde	37.60 ± 1.77de	1.04 ± 0.034c	3.11 ± 0.013def	2.305 ± 0.071ab	4.949 ± 0.551ab	2.65 ± 0.06ab	1.97 ± 0.05abc	352.28 ± 17.61efg	492.06 ± 23.40a	286.29 ± 12.92abc	303.05 ± 6.46cdefghi

*Values are expressed as mean ± SD (n = 3), and different letters indicate a significant difference (p ≤ 0.05)*.

### Antioxidant Enzyme Activity (POD and CAT)

The peroxidase activity in the mungbean microgreens, when grown at Delhi (266.17–355.41 U/g of FW), was significantly less than those of Leh-grown microgreens (307.37–518.8 U/g of FW). Similarly, peroxidase activity was recorded more for the Leh-grown lentil microgreens (401.53–502.80 U/g of FW) than the Delhi samples (335.33–437.59 U/g of FW) (Tables 3, 4). The POD activity in the lentil microgreens was found much higher than that reported in 5-day-old lentil sprouts (139.17 U/g of FW) (Swieca and Gwalik-Dziki, 2015). However, POD activity to the tune of 596.67 (U/g FW) has been recorded in the 2-day-old lentil sprouts when given continuous elicitation with 150 mM H_2_O_2_ (Swieca and Gwalik-Dziki, 2015).

CAT is the most efficient enzyme, which neutralizes H_2_O_2_ into H_2_O and O_2_, and its Kcat was the highest among all the antioxidant enzymes (Singh et al., 2015). Large variations were observed for the CAT activities of both mungbean and lentil microgreens when grown either at Leh or Delhi conditions. No specific trend has been recorded for the mungbean microgreens when grown at Delhi (239.09–467.51 U/g FW) or Leh conditions (275.63–470.56 U/g FW). Similar observations were also recorded for the lentil microgreens when grown at Delhi (159.9–319.80 U/g FW) or Leh conditions (217.77–435.53 U/g FW) (Tables 3, 4). Nearly similar CAT activity (444.00 U/g of FW) was reported in the 5-day-old lentil sprouts (Swieca and Gwalik-Dziki, 2015).

### Correlation Studies and Cluster Analysis

The correlation of measured values between total AoA (H_2_O_2_, FRAP, DPPH activity), CAT, POD, TPC, TFC, TTC, TCC, and TAA was studied using Pearson's correlation method (Table 5). In mungbean, TPC was found positively correlated with FRAP (*P* <0.01) and DPPH (*P* <0.05), while in lentil, TFC was found positively correlated with DPPH (*P* <0.01). Interestingly, FRAP and DPPH activity was also found positively correlated (*P* <0.01) in mungbean, while TCC and TAA in lentil microgreens (*P* <0.01). Also, a significant (*P* <0.05) negative correlation has been observed between CAT and TCC in mungbean microgreens. DPPH having a significantly positive correlation with FRAP (*r* = 0.966) was also reported by Fidrianny et al. (2018) in sweet potatoes. This means the AoA of samples is linearly correlated in DPPH and FRAP methods. Thus, these assays indicate that phenolic compounds (in mungbean) and flavonoid content (in lentils) are mainly contributing to the antioxidant activities, which is in tune with the previous studies (Bhoyar et al., 2011, 2018; Fidrianny et al., 2018). Interestingly, TAA was also found significantly correlated with FRAP (*r* = 0.501; *P* <0.05) and total carotenoid content (*r* = 0.704; *P* <0.01) in lentil microgreens, indicating their role in the total AoA of lentil microgreens. Such correlation studies can give a more precise idea of the parameter(s) contributing to the AoA (Patel et al., 2016; Bhalani et al., 2019; Devi et al., 2019).

**Table 5:** Correlation between various antioxidant and enzymatic parameters in (a) Mungbean and (b) Lentil microgreens.

**(a) Mungbean**	**DPPH**	**FRAP**	**H_**2**_O_**2**_**	**CAT**	**POD**	**TPC**	**TFC**	**TTC**	**TCC**	**TAA**
DPPH	1	0.709**	0.036	0.473*	0.178	0.532*	0.28	−0.202	0.353	0.371
FRAP		1	−0.052	0.574**	−0.195	0.888**	0.206	−0.391	0.273	0.388
H_2_O_2_			1	−0.188	0.178	0.128	0.383	0.269	0.262	−0.374
CAT				1	−0.085	0.367	0.436	−0.563**	0.244	0.200
POD					1	−0.308	0.219	−0.098	0.334	0.272
TPC						1	0.124	−0.321	0.135	0.184
TFC							1	−0.02	0.312	−0.014
TTC								1	−0.159	−0.231
TCC									1	0.397
TAA										1
**(b) Lentil**
DPPH	1	−0.115	−0.213	0.231	0.029	−0.342	0.909**	0.309	0.233	0.066
FRAP		1	0.385	0.079	0.169	−0.033	−0.045	0.304	0.236	0.501*
H_2_O_2_			1	−0.203	0.007	−0.206	−0.076	0.158	0.229	0.445*
CAT				1	−0.182	−0.258	0.224	0.004	−0.201	−0.141
POD					1	0.117	−0.115	−0.049	0.354	0.486*
TPC						1	−0.363	−0.085	0.295	0.137
TFC							1	0.21	0.146	0.011
TTC								1	0.414	0.489*
TCC									1	0.704**
TAA										1

(**) Correlation is significant at P ≤ 0.01; (*) at P <0.05 level. TPC, total phenolics content; TFC, total flavonoids content; TCC, total carotenoids content; TTC, total tocopherols content; TAA, total ascorbic acid.

**Figure 2:**
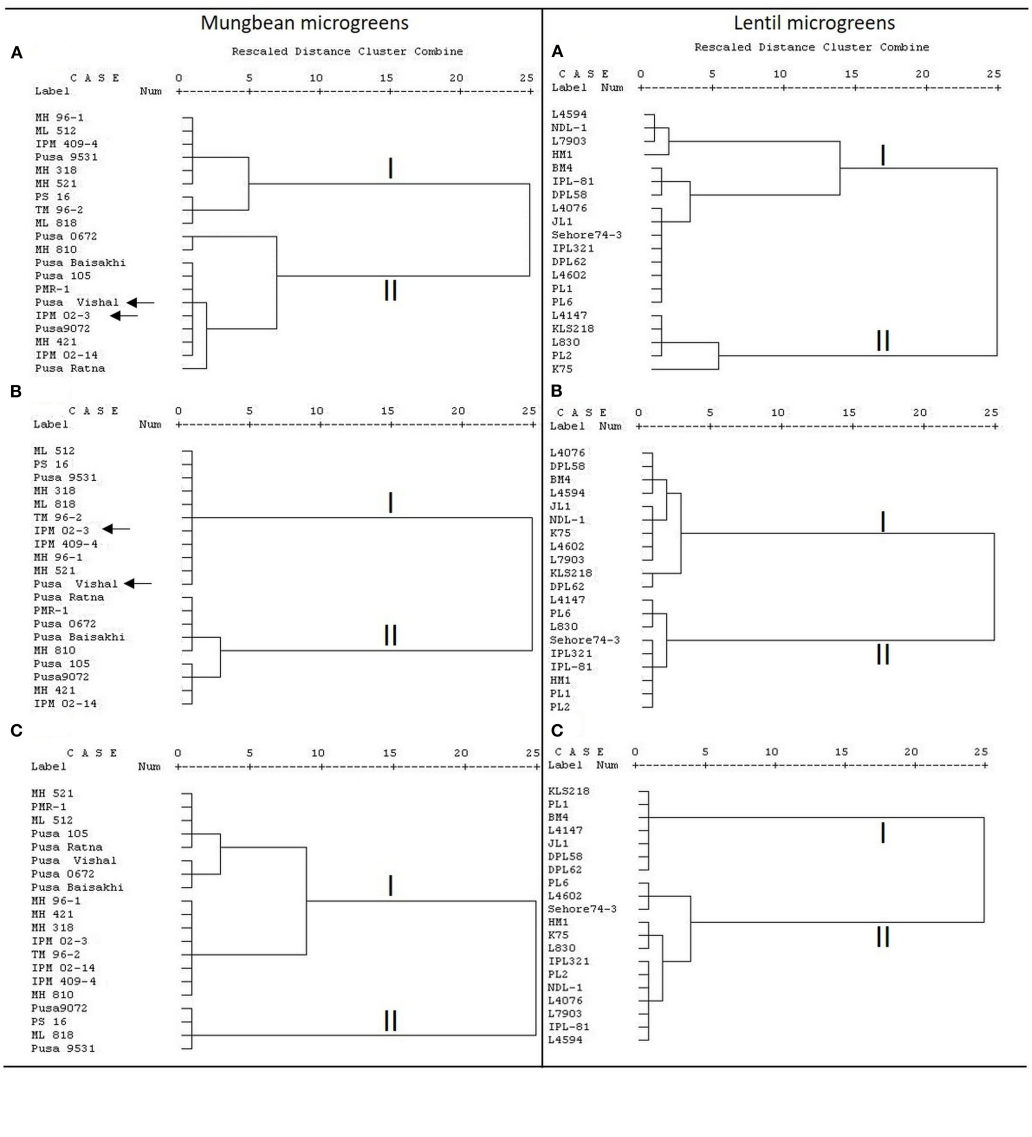
Cluster analysis based on **(A)** total AoA as measured by DPPH, ferric-reducing antioxidant power (FRAP), and peroxide assay; **(B)** total phenolics content (TPC); **(C)** total flavonoids content (TFC) in mungbean and lentil microgreens, where dendrogram was prepared using the Ward method.

Furthermore, the clusters based on total AoA and TPC are found very similar for mungbean microgreens (Figures 2A,B); and of two major clusters except for Pusa Vishal and IPM02-3, all other genotypes grouped similarly. However, for the lentil microgreens, the clusters based on total AoA, TFC, and TPC did not show any similarity in the grouping of genotypes (Figures 2A–C). In lentils, several parameters contribute to the total AoA; while in mungbean, TPC is primarily involved in imparting the total AoA. A highly positive relationship between total phenols and AoA appears to be the trend in many plant species (Oktay et al., 2003). Phenolics play a crucial role in total bioactive activities (Pereira et al., 2009). A similar trend was recorded by Bhoyar et al. (2011) for the antioxidant activities of caper leaves which were collected from different altitudes of Ladakh (India).

### PCA of Functional Characteristics of Lentil and Mungbean Microgreens

**Figure 3:**
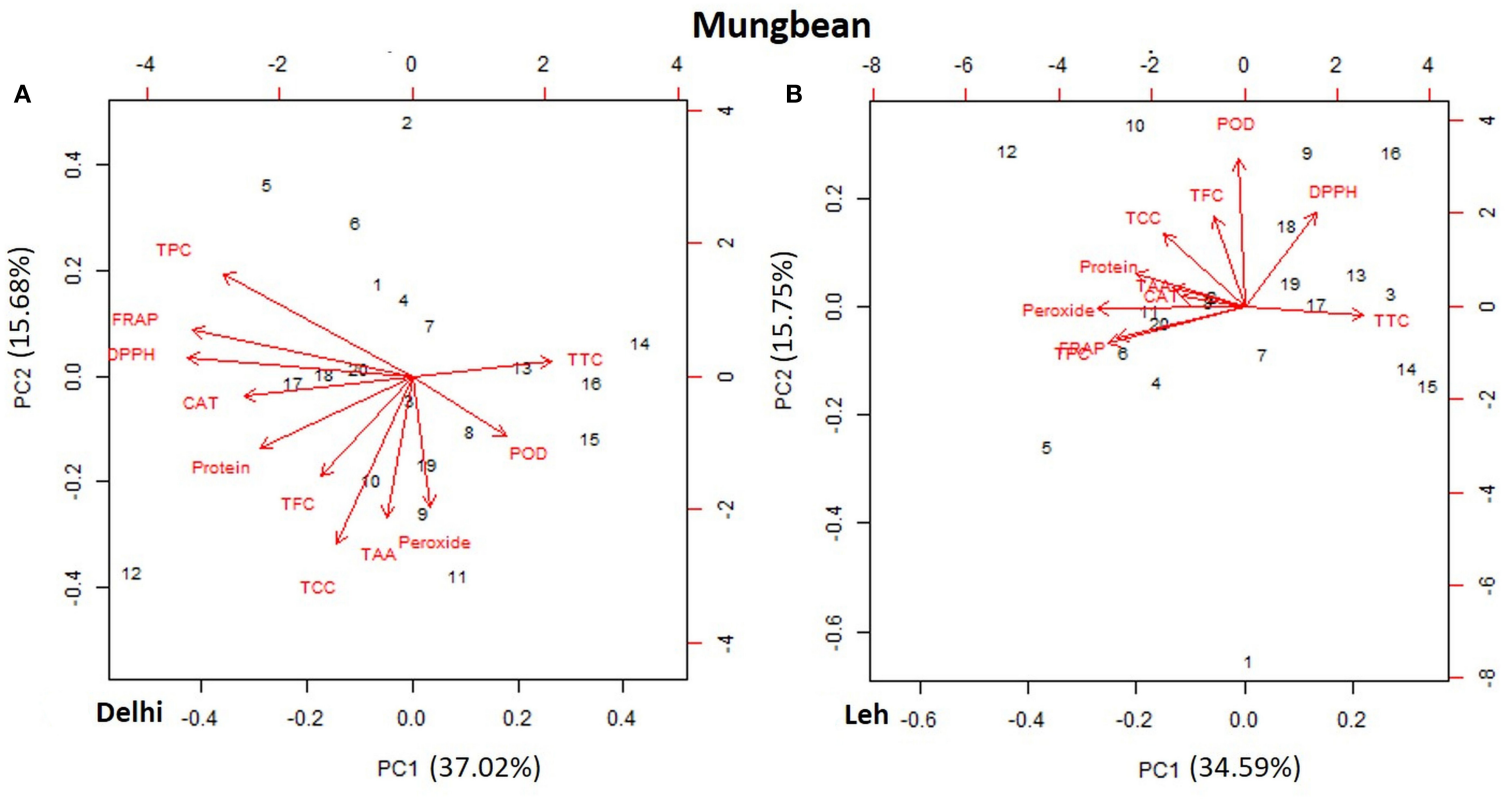
Principal component plot derived from various antioxidant activities [1,1-diphenyl-2-picrylhydrazyl (DPPH), ferric-reducing antioxidant power (FRAP), peroxide, total carotenoids content (TCC), total tocopherol content (TTC), total ascorbic acid (TAA), peroxidase (POD), and catalase (CAT)], protein, phenolics TPC, and TFC in the mungbean microgreens when grown at **(A)** normal altitude (Delhi) and **(B)** high altitude (Leh) conditions.

**Figure 4:**
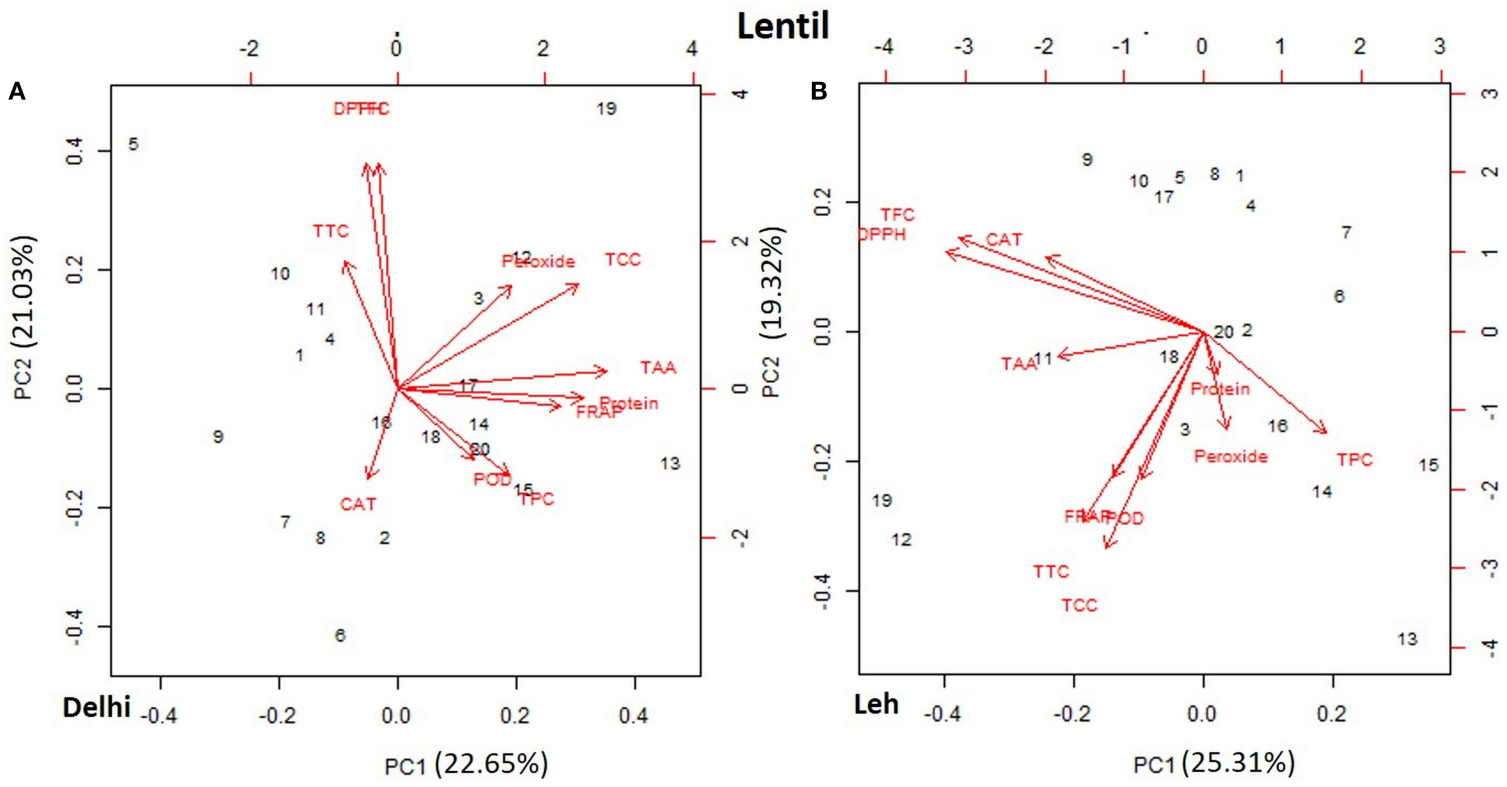
Principal component plot derived from various antioxidant activities [1,1-diphenyl-2-picrylhydrazyl (DPPH), ferric-reducing antioxidant power (FRAP), peroxide, total tocopherols content (TTC), total carotenoids content (TCC), total ascorbic acid (TAA), peroxidase (POD), and catalase (CAT)], TPC, and TFC contents in the lentil microgreens when grown at **(A)** normal altitude (Delhi) and **(B)** high altitude (Leh) conditions.

The PCA of various AoA, protein, and polyphenols of mungbean and lentil microgreens, when grown at Delhi and Leh conditions, is presented in Figures 3, 4. For mungbean microgreens when grown at Delhi and Leh, the first two principal components (PCs) explained 52.7 and 50.34% of the total variance, with PC1 and PC2 accounting for 37.02 and 15.68% and 34.59 and 15.75% of the variance, respectively (Figures 3A,B). However, for lentil microgreens when grown at Delhi and Leh, the first two PCs explained 43.68 and 44.63% of the total variance, with PC1 and PC2 accounting for 22.65 and 21.03%, and 25.31 and 19.32% of the variance, respectively (Figures 4A,B). Similarly, El-Nakhel et al. (2020) reported that the first two PCs explained 99.1% of the total variance for lettuce microgreens. The usefulness of PCA in understanding the variations at the species level across various attributes in response to varied factors like genetic makeup, maturity duration, grow-conditions, etc., have been reported by various workers (El-Nakhel et al., 2019a,b; Kyriacou et al., 2019). This study was conducted under partially controlled conditions, where growth parameters were relatively stable for Delhi conditions, while for Leh conditions, a bit harsh conditions prevailed. In addition, species-level variations are also observed in this study for lentil and mungbean species.

### Macro and Micronutrient Analysis

The macro (Ca, K, Mg, P, Na) and micro-nutrient contents (Fe, Zn, Cu, Mn) of 20 genotypes each of mungbean and lentil microgreens (expressed on a FW basis), when grown under Delhi and Leh conditions are presented in Tables 6–9. The most abundant elements in all the samples were, in the order, K, P, and Ca (mungbean) and K, Ca, and P (in lentil). Other studies also report that K is the main element accumulated in several microgreens (Wang et al., 2016; Paradiso et al., 2018). Except for K (239–369 mg/100 g FW), most of the other macro-nutrients (Ca: 47–87; Mg: 36–56; P: 66–87; Na: 30–60 mg/100 g FW) were recoded significantly more in Delhi-grown mungbean microgreens than the Leh-grown microgreens (K: 323–475; Ca: 42–57; Mg: 36–56; P: 39–67; Na: 20–36 mg/100 g FW). However, in lentil microgreens all the macronutrient contents were more for the Delhi (Ca: 45–70; K: 299–389; Mg: 29–66; P: 32–46; Na: 27–45 mg/100 g FW) over Leh-grown microgreens (Ca: 32–58; K: 217–369; Mg: 25–38; P: 63–88; Na: 31–42 mg/100 g FW). Interestingly, Ca and Mg were recorded more in lettuce microgreens over mature lettuce leaves (El-Nakhel et al., 2020). Also, an increase in K and Na content was recorded in various microgreens species like amaranth, cress, mizuna, and purslane when monochromatic red and blue lights were applied over normal light conditions (Kyriacou et al., 2019).

**Table 6:** Micro-nutrient content in various mungbean microgreens grown at Delhi and Leh conditions.

**S. No**.	**Genotype (Mungbean)**	**Fe (mg/100 g FW)**		**Zn (mg/100 g FW)**		**Cu (mg/100 g FW)**		**Mn (mg/100 g FW)**	
		**Delhi**	**Leh**	**Delhi**	**Leh**	**Delhi**	**Leh**	**Delhi**	**Leh**
1	Pusa Baisakhi	0.50 ± 0.01de	0.76 ± 0.07abc	0.24 ± 0.014abcd	0.29 ± 0.015abcd	0.06 ± 0.0011d	0.09 ± 0.0015ab	0.15 ± 0.003defg	0.14 ± 0.008bcd
2	Pusa Ratna	0.56 ± 0.012b	0.77 ± 0.04abc	0.23 ± 0.01abcde	0.25 ± 0.009efgh	0.06 ± 0.001d	0.08 ± 0.002c	0.17 ± 0.004abcd	0.15 ± 0.003ab
3	Pusa Vishal	0.46 ± 0.012fgh	0.71 ± 0.06abcdefg	0.21 ± 0.01cde	0.25 ± 0.012defgh	0.07 ± 0.0011c	0.05 ± 0.0016e	0.14 ± 0.005efg	**0.09** **±** **0.005g**
4	Pusa105	0.50 ± 0.01de	0.74 ± 0.04abcde	0.25 ± 0.012ab	0.24 ± 0.01efghj	0.08 ± 0.0012c	0.08 ± 0.008c	0.14 ± 0.005defg	0.18 ± 0.008a
5	Pusa0672	0.56 ± 0.015b	0.70 ± 0.01abcdefg	0.23 ± 0.015abcde	0.26 ± 0.009cdef	0.05 ± 0.0006ef	0.09 ± 0.0014b	**0.09** **±** **0.005j**	0.16 ± 0.005ab
6	Pusa9072	0.49 ± 0.006def	0.70 ± 0.02abcdefg	0.26 ± 0.01a	0.25 ± 0.012efgh	0.07 ± 0.0008c	0.08 ± 0.008c	0.16 ± 0.006bcde	0.16 ± 0.006ab
7	Pusa9531	0.48 ± 0.01efg	0.64 ± 0.03efg	0.21 ± 0.011cde	**0.21** **±** **0.006h**	0.08 ± 0.0022c	0.05 ± 0.0016e	0.18 ± 0.008ab	0.10 ± 0.011fg
8	MH96-1	0.52 ± 0.015cd	0.78 ± 0.02abc	0.26 ± 0.01a	0.30 ± 0.017ab	0.05 ± 0.0007e	0.10 ± 0.009a	0.18 ± 0.008ab	0.13 ± 0.009cde
9	MH318	0.42 ± 0.009ij	0.65 ± 0.02efg	0.24 ± 0.01abc	0.31 ± 0.009ab	0.06 ± 0.0004d	0.06 ± 0.001d	0.10 ± 0.007ij	0.13 ± 0.002cde
10	MH421	0.53 ± 0.015bc	0.72 ± 0.02abcdefg	0.21 ± 0.016cde	0.22 ± 0.008gh	**0.03** **±** **0.0006g**	**0.04** **±** **0.0017f**	0.10 ± 0.003ij	**0.18** **±** **0.008a**
11	MH521	**0.59** **±** **0.011a**	0.68 ± 0.02cdefg	0.21 ± 0.004de	0.29 ± 0.015abcd	0.04 ± 0.0017f	0.09 ± 0.0058ab	0.11 ± 0.002hij	0.16 ± 0.006ab
12	**MH810**	0.52 ± 0.015cd	***0.76±0.01abcd***	0.22 ± 0.008bcde	0.25 ± 0.013efgh	0.05 ± 0.001e	0.08 ± 0.0022c	0.14 ± 0.008efg	***0.15±0.003ab***
13	ML512	0.50 ± 0.01de	0.63 ± 0.03g	0.22 ± 0.1bcde	0.28 ± 0.007bcde	0.09 ± 0.0004b	0.09 ± 0.009cb	0.16 ± 0.005bcde	0.16 ± 0.005ab
14	ML818	**0.40** **±** **0.011j**	0.74 ± 0.03abcdef	0.21 ± 0.015de	0.24 ± 0.014fgh	0.09 ± 0.001b	0.08 ± 0.0022c	0.18 ± 0.007ab	0.13 ± 0.002cde
15	PS16	0.48 ± 0.008efg	0.63 ± 0.01fg	**0.20** **±** **0.010e**	**0.32** **±** **0.019a**	0.05 ± 0.0016e	0.09 ± 0.0016b	0.10 ± 0.011ij	0.12 ± 0.005cdef
16	TM96-2	0.51 ± 0.004cde	0.69 ± 0.02bcdefg	**0.26** **±** **0.009a**	0.29 ± 0.013abc	0.03 ± 0.0014g	0.06 ± 0.0012d	0.16 ± 0.007bcde	0.11 ± 0.002efg
17	IPM02-3	0.44 ± 0.01hi	**0.79** **±** **0.02a**	0.22 ± 0.012bcde	0.25 ± 0.015cdefg	0.08 ± 0.0008c	**0.10** **±** **0.009a**	0.13 ± 0.002fgh	0.12 ± 0.004def
18	IPM02-14	0.52 ± 0.011cd	**0.62** **±** **0.02g**	0.21 ± 0.006cde	0.28 ± 0.007bcde	0.06 ± 0.0012d	0.09 ± 0.001b	0.13 ± 0.009fgh	0.13 ± 0.007cde
19	IPM409-4	0.43 ± 0.011ij	0.68 ± 0.01cdefg	0.23 ± 0.006bcde	0.24 ± 0.01efgh	0.08 ± 0.0002c	0.06 ± 0.004d	0.17 ± 0.014abc	0.12 ± 0.009cdef
20	PMR-1	0.45 ± 0.011ghi	0.65 ± 0.01defg	0.22 ± 0.008bcde	0.29 ± 0.008abc	**0.10** **±** **0.0009a**	0.09 ± 0.0027ab	**0.19** **±** **0.013a**	0.14 ± 0.003bc

*Values are expressed as mean ± SD (n = 3), and different letters indicate a significant difference (p ≤ 0.05)*.

**Table 7:** Micro-nutrient content in various lentil microgreens grown at Delhi and Leh conditions.

**S. No**.	**Genotype (Lentil)**	**Fe (mg/100 g FW)**		**Zn (mg/100 g FW)**		**Cu (mg/100 g FW)**		**Mn (mg/100 g FW)**	
		**Delhi**	**Leh**	**Delhi**	**Leh**	**Delhi**	**Leh**	**Delhi**	**Leh**
1	L4076	0.66 ± 0.01abc	0.66 ± 0.031defg	0.34 ± 0.007b	0.39 ± 0.009abcdefg	**0.18** **±** **0.007a**	0.20 ± 0.027ab	0.15 ± 0.003a	0.13 ± 0.01abcde
2	L4147	0.53 ± 0.095efgh	0.61 ± 0.02fgh	0.40 ± 0.008a	**0.26** **±** **0.017i**	0.06 ± 0.002efg	0.14 ± 0.036bcdef	0.10 ± 0.009c	0.18 ± 0.036a
3	L4594	0.61 ± 0.02cde	0.65 ± 0.01efgh	0.39 ± 0.009ab	0.37 ± 0.024defgh	0.12 ± 0.002bc	0.13 ± 0.031bcdef	0.10 ± 0.009c	0.13 ± 0.031abcde
4	L7903	0.57 ± 0.025cdef	0.75 ± 0.032ab	0.40 ± 0.013ab	0.40 ± 0.034abcdefg	**0.05** **±** **0.002g**	0.09 ± 0.011ef	0.08 ± 0.004d	0.09 ± 0.011de
5	HM1	0.55 ± 0.032defg	0.72 ± 0.018bcd	0.39 ± 0.016ab	0.28 ± 0.041hi	0.05 ± 0.002g	0.12 ± 0.017cdef	0.08 ± 0.004d	0.14 ± 0.036abcde
6	BM4	0.64 ± 0.042bcd	0.79 ± 0.01a	0.38 ± 0.006ab	0.32 ± 0.02ghi	0.07 ± 0.010ef	0.16 ± 0.010abcd	0.10 ± 0.009c	**0.18** **±** **0.035a**
7	JL1	0.51 ± 0.010efgh	0.69 ± 0.015cde	0.38 ± 0.015ab	0.46 ± 0.028abcd	0.06 ± 0.001fg	0.19 ± 0.020ab	0.11 ± 0.003bc	0.17 ± 0.010abc
8	Sehore74-3	0.50 ± 0.04fgh	0.64 ± 0.031efgh	0.39 ± 0.056ab	0.49 ± 0.015ab	0.06 ± 0.001fg	0.18 ± 0.032abc	0.08 ± 0.004d	0.17 ± 0.023ab
9	NDL-1	0.46 ± 0.025gh	0.74 ± 0.036abc	0.37 ± 0.010ab	0.39 ± 0.057bcdefg	0.08 ± 0.005e	**0.22** **±** **0.028a**	0.10 ± 0.001c	0.13 ± 0.031abcde
10	IPL81	0.56 ± 0.029cdefg	0.68 ± 0.017de	0.39 ± 0.009ab	0.47 ± 0.058abc	0.13 ± 0.004b	0.10 ± 0.001def	0.12 ± 0.02b	0.13 ± 0.030abcde
11	IPL321	0.59 ± 0.040cdef	0.59 ± 0.020h	**0.40** **±** **0.025a**	0.33 ± 0.037fghi	0.06 ± 0.006efg	**0.09** **±** **0.001f**	0.10 ± 0.004c	0.09 ± 0.016de
12	K75	0.74 ± 0.041ab	**0.79** **±** **0.015a**	**0.23** **±** **0.020c**	0.49 ± 0.010a	0.10 ± 0.001d	0.15 ± 0.030bcdef	0.12 ± 0.006b	0.12 ± 0.017abcde
13	KLS218	0.55 ± 0.039defgh	0.65 ± 0.001efgh	0.39 ± 0.009ab	0.44 ± 0.038abcde	0.11 ± 0.009cd	0.10 ± 0.009def	**0.15** **±** **0.004a**	0.11 ± 0.003bcde
14	DPL58	0.59 ± 0.010cdef	0.60 ± 0.023gh	0.38 ± 0.029ab	0.34 ± 0.037efghi	0.05 ± 0.002g	0.10 ± 0.009def	0.08 ± 0.005de	0.08 ± 0.003e
15	DPL62	**0.45** **±** **0.006h**	0.73 ± 0.021abcd	0.26 ± 0.033c	0.42 ± 0.068abcdef	0.11 ± 0.003cd	0.09 ± 0.002ef	0.10 ± 0.009c	**0.08** **±** **0.006e**
16	PL1	0.55 ± 0.001defgh	0.68 ± 0.017cde	0.24 ± 0.019c	0.37 ± 0.010cdefgh	0.12 ± 0.004b	0.16 ± 0.019abcde	0.08 ± 0.005de	0.17 ± 0.010abc
17	PL2	0.58 ± 0.020cdef	0.67 ± 0.001bef	0.25 ± 0.013c	0.48 ± 0.030abc	0.13 ± 0.009b	0.13 ± 0.010bcdef	**0.06** **±** **0.001e**	0.15 ± 0.030abcd
18	PL6	0.59 ± 0.020cdef	**0.52** **±** **0.017i**	0.40 ± 0.015ab	0.36 ± 0.007defghi	0.06 ± 0.001fg	0.18 ± 0.035abc	0.10 ± 0.009c	0.15 ± 0.030abcd
19	**L830**	0.59 ± 0.004cdef	***0.76±0.010ab***	0.23 ± 0.004c	***0.49±0.015a***	0.11 ± 0.002cd	***0.19±0.024abc***	0.12 ± 0.006b	0.10 ± 0.021cde
20	L4602	**0.75** **±** **0.032a**	0.79 ± 0.035ab	0.37 ± 0.001ab	0.45 ± 0.010abcd	0.07 ± 0.010ef	0.17 ± 0.003abc	0.10 ± 0.009c	0.17 ± 0.010abc

*Values are expressed as mean ± SD (n = 3), and different letters indicate a significant difference (p ≤ 0.05)*.

**Table 8:** Macro-nutrient content in various mungbean microgreens grown at Delhi and Leh conditions.

**S. No**.	**Genotype (Mungbean)**	**Ca (mg/100 g FW)**		**K (mg/100 g FW)**		**Mg (mg/100 g FW)**		**P (mg/100 g FW)**		**Na (mg/100 g FW)**	
		**Delhi**	**Leh**	**Delhi**	**Leh**	**Delhi**	**Leh**	**Delhi**	**Leh**	**Delhi**	**Leh**
	Pusa Baisakhi	65 ± 2.0bcd	50 ± 1.73bcde	291 ± 4.4fghi	420 ± 6.60cd	56 ± 3.0a	43 ± 3.6abc	74 ± 1.00ef	45 ± 2.6cdef	40 ± 3.00c	36 ± 0.5a
	Pusa Ratna	51 ± 2.0fg	45 ± 1.00defg	303 ± 10.8defgh	429 ± 2.60cd	49 ± 1.00b	30 ± 1.0fg	**87** **±** **2.00a**	40 ± 1.0ef	30 ± 1.00d	23 ± 1.7efg
	Pusa Vishal	47 ± 2.0g	44 ± 2.00efg	**239** **±** **8.2 l**	**354** **±** **5.30e**	40 ± 2.00de	41 ± 2.0abcde	71 ± 1.73fgh	51 ± 2.6bc	30 ± 1.71d	20 ± 0.06g
	Pusa105	81 ± 2.6a	53 ± 2.65abc	342 ± 9.6abc	467 ± 2.00ab	52 ± 2.00ab	35 ± 1.0cdefg	81 ± 2.65bc	41 ± 1.7def	30 ± 1.00d	**36** **±** **1.1a**
	Pusa0672	51 ± 2.0fg	45 ± 2.00defg	281 ± 7.5ghij	445 ± 5.60bc	48 ± 1.73bc	33 ± 2.6efg	75 ± 1.73def	45 ± 3.5cdef	30 ± 1.72d	24 ± 1.8efg
	Pusa9072	60 ± 2.6de	45 ± 1.00defg	325 ± 12.1cde	438 ± 8.90cd	44 ± 2.00cd	42 ± 4.0abcd	83 ± 2.00ab	44 ± 3.6cdef	50 ± 4.58b	26 ± 1.0def
	Pusa9531	66 ± 2.2bcd	42 ± 1.73fg	320 ± 8.7cde	323 ± 9.80f	50 ± 1.73b	31 ± 1.0fg	81 ± 2.65bc	42 ± 3.0def	60 ± 2.65a	22 ± 0.5fg
	MH96-1	60 ± 1.0de	46 ± 1.00defg	**369** **±** **14.9a**	436 ± 4.40cd	48 ± 1.00bc	30 ± 4.6fg	68 ± 1.73gh	43 ± 1.7def	**30** **±** **2.00d**	30 ± 2.0bcd
	MH318	**47** **±** **2.0g**	**40** **±** **1.00g**	251 ± 8.5kl	423 ± 7.50cd	42 ± 1.00d	35 ± 2.0cdefg	84 ± 1.73ab	47 ± 3.0cde	50 ± 3.00b	31 ± 2.0bc
	MH421	67 ± 1.0 bc	54 ± 2.00ab	309 ± 4.6def	435 ± 10.60cd	50 ± 1.00b	37 ± 2.6bcdef	77 ± 1.73cde	45 ± 2.6cdef	50 ± 4.00b	33 ± 1.5ab
	MH521	60 ± 3.0de	**57** **±** **3.61a**	250 ± 9.0kl	430 ± 2.60cd	50 ± 1.73b	40 ± 3.5abcde	77 ± 1.00cde	46 ± 1.0cde	30 ± 1.00d	29 ± 1.0bcd
	**MH810**	54 ± 2.0ef	47 ± 2.65cdef	258 ± 9.8jkl	***473±8.90a***	44 ± 1.00cd	34 ± 1.0defg	75 ± 2.65def	**38** **±** **1.7f**	30 ± 1.7d	21 ± 1.9g
	ML512	70 ± 1.0b	51 ± 1.73abcd	304 ± 11.5defg	427 ± 10.80cd	48 ± 1.00bc	40 ± 2.6abcde	81 ± 2.00bc	42 ± 2.0def	40 ± 2.65c	21 ± 1.0g
	ML818	70 ± 2.6b	53 ± 2.00abc	330 ± 12.0bcd	**475** **±** **6.00a**	42 ± 2.00d	34 ± 1.0defg	80 ± 3.00bcd	41 ± 2.6def	30 ± 1.00d	32 ± 1.5ab
	PS16	68 ± 2.0bc	56 ± 2.65ab	267 ± 7.5ijk	377 ± 9.80e	51 ± 1.73b	45 ± 4.4ab	**66** **±** **2.00h**	43 ± 3.0def	60 ± 5.00a	27 ± 1.2cde
	TM96-2	**86** **±** **2.6a**	42 ± 1.00fg	357 ± 5.2ab	435 ± 11.10cd	49 ± 2.60b	36 ± 2.0cdefg	73 ± 1.73efg	56 ± 3.5b	30 ± 1.74d	32 ± 0.9ab
	IPM02-3	69 ± 1.7b	47 ± 1.00cdef	276 ± 7.2hijk	415 ± 12.00d	**36** **±** **1.73e**	**28** **±** **1.7g**	76 ± 1.00cdef	44 ± 2.6cdef	**60** **±** **5.57a**	21 ± 1.0g
	IPM02-14	62 ± 2.0cd	50 ± 3.00bcde	299 ± 6.6efgh	369 ± 9.20e	49 ± 1.73b	45 ± 2.0ab	72 ± 2.00efg	**67** **±** **1.7a**	60 ± 2.00a	**20** **±** **0.7g**
	IPM409-4	67 ± 2.6bc	50 ± 2.00bcde	256 ± 7.2jkl	419 ± 8.00d	**56** **±** **2.60a**	**46** **±** **3.0a**	72 ± 2.65efg	48 ± 2.0cd	40 ± 2.65c	31 ± 1.0bc
	PMR-1	67 ± 1.0bc	42 ± 1.00fg	259 ± 6.2jkl	428 ± 10.00cd	41 ± 2.00b	42 ± 1.7abcd	72 ± 1.00efg	45 ± 3.6cdef	60 ± 4.58a	31 ± 1.7bc

*Values are expressed as mean ± SD (n = 3), and different letters indicate a significant difference (p ≤ 0.05)*.

**Table 9:** Macro-nutrient content in various lentil microgreens grown at Delhi and Leh conditions.

**S. No**.	**Genotype (Lentil)**	**Ca (mg/100 g FW)**		**K (mg/100 g FW)**		**Mg (mg/100 g FW)**		**P (mg/100 g FW)**		**Na (mg/100 g FW)**	
		**Delhi**	**Leh**	**Delhi**	**Leh**	**Delhi**	**Leh**	**Delhi**	**Leh**	**Delhi**	**Leh**
1	L4076	50 ± 3.61de	40 ± 3.61defg	**389** **±** **12.77a**	272 ± 8.89cd	35 ± 1.00fg	33 ± 1.00abc	34 ± 3.00ab	64 ± 2.65fg	33 ± 4.36bcd	32 ± 1.89ab
2	L4147	62 ± 3.46abc	56 ± 3.61ab	374 ± 13.23ab	364 ± 7.94a	47 ± 4.58bcde	31 ± 3.46abc	35 ± 3.60ab	69 ± 4.36defg	32 ± 1.76bcd	40 ± 4.58ab
3	L4594	57 ± 5.00bcd	58 ± 2.65a	317 ± 10.00def	234 ± 6.56fg	**32** **±** **2.00g**	28 ± 2.00bc	45 ± 3.61ab	70 ± 5.29defg	32 ± 1.70bcd	35 ± 3.46ab
4	L7903	51 ± 1.73de	55 ± 4.36abc	374 ± 17.32ab	230 ± 6.56fg	51 ± 4.36b	31 ± 4.00abc	37 ± 2.00ab	71 ± 2.00defg	33 ± 4.36bcd	32 ± 1.72ab
5	HM1	48 ± 2.00de	**57** **±** **4.00a**	348 ± 13.45bcde	235 ± 6.08fg	47 ± 3.00bcde	36 ± 3.00ab	38 ± 8.72ab	69 ± 3.61defg	29 ± 2.00cd	41 ± 1.73ab
6	BM4	49 ± 4.36de	49 ± 2.00abcd	373 ± 25.51ab	292 ± 7.21c	37 ± 2.00efg	30 ± 2.00abc	37 ± 4.58ab	68 ± 5.00defg	**25** **±** **1.00d**	42 ± 3.00a
7	JL1	**70** **±** **5.29a**	36 ± 4.36g	319 ± 8.91cdef	254 ± 5.00def	51 ± 1.73b	26 ± 3.00c	33 ± 4.36ab	87 ± 6.24ab	33 ± 4.36bcd	32 ± 1.56ab
8	Sehore74-3	53 ± 3.00cde	36 ± 3.61g	364 ± 9.71ab	288 ± 10.00c	49 ± 4.00bc	36 ± 2.65ab	36 ± 3.61ab	66 ± 4.58efg	28 ± 4.58cd	38 ± 3.61ab
9	NDL-1	50 ± 2.65de	49 ± 2.00abcd	353 ± 11.00abcd	**369** **±** **11.79a**	43 ± 2.65bcdef	28 ± 2.65bc	33 ± 1.00ab	76 ± 2.00bcde	29 ± 2.65cd	36 ± 1.11ab
10	IPL81	52 ± 2.65cde	**35** **±** **1.00g**	**299** **±** **16.46f**	239 ± 7.55efg	36 ± 2.00fg	33 ± 4.58abc	37 ± 6.56ab	75 ± 2.65cdef	30 ± 2.65bcd	39 ± 4.58ab
11	IPL321	67 ± 6.24ab	46 ± 2.65cdef	361 ± 9.00ab	255 ± 12.29def	38 ± 3.61defg	**25** **±** **3.61c**	43 ± 5.25ab	77 ± 1.73abcde	34 ± 5.20bcd	39 ± 5.24ab
12	K75	58 ± 1.73bcd	32 ± 1.73g	363 ± 5.28ab	254 ± 10.00def	48 ± 4.58bcd	29 ± 2.05bc	37 ± 5.22ab	74 ± 3.61cdefg	34 ± 2.00bcd	**42±3.00**a
13	KLS218	66 ± 2.00ab	47 ± 5.20bcde	367 ± 10.00ab	253 ± 7.94def	**66** **±** **4.58a**	29 ± 1.76bc	36 ± 2.00ab	78 ± 3.00abcd	29 ± 1.00cd	38 ± 3.61ab
14	DPL58	53 ± 1.73cde	46 ± 2.65cdef	359 ± 12.49abc	271 ± 8.00cd	50 ± 2.65b	27 ± 1.00c	38 ± 5.00ab	77 ± 1.00abcde	27 ± 3.61d	37 ± 5.21ab
15	DPL62	51 ± 3.61de	47 ± 2.65bcde	312 ± 7.21ef	247 ± 1.54defg	34 ± 3.00fg	30 ± 2.65abc	42 ± 4.36ab	71 ± 2.65defg	**45** **±** **3.61a**	37 ± 5.29ab
16	PL1	57 ± 4.00bcd	38 ± 3.61efg	351 ± 10.00abcde	269 ± 7.55cde	29 ± 2.00g	31 ± 1.74abc	**46** **±** **4.58a**	**63** **±** **2.65g**	39 ± 2.00ab	36 ± 0.50ab
17	PL2	**45** **±** **2.00e**	36 ± 1.00g	352 ± 14.00abcde	**217** **±** **10.00g**	31 ± 5.25g	29 ± 1.95bc	**32** **±** **1.73b**	**88** **±** **4.58a**	34 ± 1.00bcd	39 ± 3.00ab
18	PL6	69 ± 4.58a	37 ± 1.73fg	355 ± 12.5abcd	296 ± 22.11c	44 ± 4.00bcdef	27 ± 1.00c	36 ± 3.59ab	69 ± 1.00defg	37 ± 5.26abc	**31** **±** **1.00b**
19	**L830**	52 ± 2.65cde	***56±4.00ab***	***355±20.22abcd***	295 ± 6.24c	39 ± 2.00cdefg	29 ± 1.00bc	***38±2.00ab***	***85±5.29abc***	32 ± 1.72bcd	***32±1.86ab***
20	L4602	50 ± 1.73de	52 ± 2.00abc	345 ± 2.65bcde	329 ± 11.14b	48 ± 2.65bcd	**38** **±** **3.00a**	36 ± 2.00ab	75 ± 2.00cdef	33 ± 1.00bcd	37 ± 5.27ab

*Values are expressed as mean ± SD (n = 3), and different letters indicate a significant difference (p ≤ 0.05)*.

For micronutrients, the Delhi-grown mungbean microgreens showed significantly less Fe (0.4–0.59 mg/100 g FW) and Zn (0.2–0.26 mg/100 g FW) content than the Leh-grown microgreens (Fe: 0.62–0.79; Zn: 0.21–0.32 mg/100 g FW). However, no such trend was recorded for Cu and Mn content in mungbean microgreens when grown at Delhi (Cu: 0.03–0.1; Mn: 0.09–0.19 mg/100 g FW) or Leh conditions (Cu: 0.04–0.1; Mn: 0.09–0.18 mg/100 g FW). In lentil, the Fe and Cu content was found more for Leh-grown microgreens (Fe: 0.52–0.79; Cu: 0.09–0.22; mg/100 g FW) than the Delhi-grown microgreens (Fe: 0.45–0.75 Cu: 0.05–0.18; mg/100 g FW). However, no such trend was recorded for other micronutrients in the lentil microgreens when grown at Delhi (Zn: 0.23–0.4; Mn: 0.06–0.15 mg/100 g FW) or Leh conditions (Zn: 0.26–0.49; Mn: 0.08–0.18 mg/100 g FW). Overall, of the 20 mungbean and lentil genotypes studied, the majority of them showed relatively more micronutrient content at Leh over Delhi-grown microgreens (Tables 6–9).

The range of various macro-nutrients in the *Cichorium, Lactuca*, and *Brassica* derived microgreens for P (536–1,039 μg/g FW), K (2,498–4,735 μg/g FW), Ca (1,008–2,027 μg/g FW), Mg (200–252 μg/g FW), and Na (73–474 μg/g FW) (Paradiso et al., 2018) were found nearly similar to that of mungbean and lentil microgreens. Similarly, the micronutrients such as Fe (4.3–16.6 μg/g FW), Zn (3.0–5.2 μg/g FW), Mn (5.3–13.2 μg/g FW), and Cu (0.29–1.18 μg/g FW) (Paradiso et al., 2018) were also in the similar range in different microgreens as that of mungbean, and lentil derived microgreens.

Among micronutrients, Fe content was always higher than Zn, as also reported for several microgreens like *Cichorium intybus* (Fe: 14.1; Zn: 4.4 μg/g FW) and *Lactuca sativa* (Fe: 16.6; Zn 5.2 μg/g FW) (Paradiso et al., 2018). In addition, the lettuce cultivar “Trocadero” showed much higher micro-and macro-nutrient contents (P: 872; K: 4,735; Mg: 248; Fe: 16.6 μg/g FW) when compared with “Bionda da taglio” cultivar (P: 536; K: 2,865; Mg: 200; Fe: 4.3 μg/g FW) (Paradiso et al., 2018). This again reiterated the need to do a wide genotypic study to find the best genotype having more mineral contents. Moreover, wide variations in the biomolecule composition of the same genotype under Delhi and Leh conditions signify the differential expression of several genes under different environmental conditions.

### Effect of Weather Parameters

The samples under study were grown during the first week of November 2019, under Leh and Delhi conditions. Relatively larger temperature amplitude (14–30°C), total photoperiod (10.4 h), UV-B (0.2–0.3 μW/cm^2^), and PAR (600 to 800 μmol/m^2^/s) were recorded under Leh growing conditions. Whereas, under Delhi conditions, the temperature was kept in the range of 18 to 26°C (mungbean; 28/26°C and lentil 21/18°C), along with natural day and night cycles (with mean day length of nearly 10.30 h) and PAR (350–500 μmol/m^2^/s); while UV-B could not be detected during the microgreens growth period in the glasshouse (Supplementary File 1). The differential growing conditions might have created more oxidative stress at Leh, and in response, the tiny plants have over-activated their antioxidant defense mechanism at Leh over Delhi conditions. Higher AoA of the same mungbean or lentil genotype(s) at Leh over Delhi conditions seems associated with differential gene expression.

Light conditions are known to influence the morpho-physiology of microgreens significantly, and also the biosynthesis and accumulation of phytochemicals (Delian et al., 2015). A lower dose of UV-B radiation is reported causing alterations in antioxidant status, e.g., regulation of glutathione pathways, phenylpropanoids, cinnamates, or flavonoids pathways, and pyridoxine biosynthesis pathways (Hideg et al., 2013). Thus, the presence of even very little UV-B radiation seems to have triggered some defense response which is reflected in terms of higher antioxidant levels (e.g., phenols and flavonoids) in the Leh-grown microgreens (Olsson et al., 1998; Hideg et al., 2013). Similarly, Brazaityte et al. (2015a) have also shown the improved antioxidant characteristics of various microgreens like basil, beet, and red Pak Choi by applying UV irradiation. A higher level of PAR has also shown higher TPC and TFC in capsicum fruits and also more flavonoids in bean leaves than a low level of PAR (Cen and Bornman, 1990). Additionally, Kamal et al. (2020) have also shown the effect of supplemental lighting causing improved vegetative growth, mineral and vitamin contents in various brassica microgreens like Kohlrabi purple, Cabbage red, Broccoli, Kale Tucsan, Komatsuna red, Tatsoi, and Cabbage green.

Stress response in the plant is associated with the enhanced production of phenolics which act as either signaling compounds, antioxidants, and/or cell wall precursors (Swieca and Gwalik-Dziki, 2015). Furthermore, phenolics synthesis activation in plants through various elicitation mechanisms means an increased antioxidant capacity. Nearly 65% increase in the phenolics content in lentil sprouts (over control) was recorded when treated with 15 mM H_2_O_2_ (Swieca and Gwalik-Dziki, 2015). Similarly, Swieca and Baraniak (2013) reported a 1.6- and 1.9-fold increase in total antioxidant capacity of 4-day-old lentil sprouts when treated with 20 and 200 mM H_2_O_2_ at 2-day-old sprouts stage. In addition, the imposition of temperature stress at 4 and 40°C for 1 h also enhanced the phenolic content and antioxidant capacity of lentil sprouts (Swieca et al., 2014b). In lentil sprouts, the UV-B treatment has resulted in enhanced phenolic content and AoA (Swieca et al., 2014a).

In general, the mean content of total phenolics, flavonoids, carotenoids, tocopherols, and ascorbic acid was recorded more in the microgreens grown under Leh than when grown under partially controlled growing conditions of Delhi. When mungbean and lentil microgreens were compared, TPC and TCC were relatively more in the lentil-based microgreens while TFC and TAA were more in the mungbean microgreens (Tables 1, 2). Except for the crude protein content, other parameters such as FRAP, DPPH, peroxide activities, and enzymatic antioxidant activities of mungbean and lentil microgreens also recorded more for the Leh-grown microgreens than the microgreens grown under partially controlled Delhi conditions. Also, relatively more FRAP and peroxidase activities were recorded for the lentil microgreens, while protein, peroxidase, and catalase were relatively more for the mungbean microgreens (Tables 3, 4). Genetic factors and growing conditions also play an important role in the formation of secondary metabolites, including various compounds imparting AoA (Islam et al., 2003). Variation in the AoA between Leh and Delhi samples could be attributed to the variation in the growing conditions. The higher accumulation of various antioxidants in the microgreens grown at Leh could be due to the relatively harsh growing conditions at Leh than that of Delhi, which may have provided better tolerance to the microgreens by either scavenging the free oxygen radicals or by protecting the innate protein from denaturation. It seems that under Leh conditions, the tiny plants preferred to synthesize more antioxidants at the cost of protein synthesis. This needs further detailed analysis.

## Conclusion

This study has identified a wide diversity in the phytochemical composition, antioxidant capacities, and nutrient contents among the microgreens of 20 diverse genotypes, each of mungbean and lentils when grown at plain-altitude and high-altitude regions. Similarly, significant variations were also recorded for mineral, phytochemical and antioxidant capacity traits among the microgreens of two lettuce cultivars (green and red Salanova®) (El-Nakhel et al., 2020). Paradiso et al. (2018) have also studied and compared the diverse nutritional profile of two varieties each of three species viz. chicory (i.e., Molfetta and Italico a costa rossa), lettuce (i.e., Bionda da taglio and Trocadero), and broccoli (i.e., Mugnuli and Natalino).

As microgreens can be grown easily at home or under very harsh conditions of Leh–Ladakh (under greenhouse conditions), they can be considered an alternative for the nutritional security of the population living in those remote areas, especially during land-locked conditions. Among 20 lentil genotypes studied, the genotype L830 was superior for several parameters like TFC, TCC, and TAA. Even for other antioxidant parameters like TPC and TTC, L830 has expressed higher content over many genotypes. Similarly, for mungbean genotypes, MH810 was found significantly superior for TPC, TFC, TCC, and TAA. In addition, microgreens cultivation should be explored as a means for providing larger quantities of nutrients (including antioxidants) per gram plant biomass over cost-intensive air-transported mature vegetables in the high-altitude areas of Ladakh, especially during winter months (Weber, 2017). Wide variations in the microgreens phytochemical compositions of the same genotype of mungbean and lentil, when grown under Delhi and Leh conditions, suggests the need to identify the genes/pathways which change its expression under different environmental conditions. This, in the long term, will help in increasing the various phytochemical compositions of microgreens by manipulating the microgreens growing conditions. This study reiterates the need to develop a deeper understanding of the intricate regulation of various antioxidant responses of the microgreens under varied environmental conditions, including temperature amplitude, UV-B, and PAR. The nutrient composition of mungbean and lentil microgreens obtained is of considerable importance to the nutritionist, consumers, and growers to select nutrient-rich, easily available, and cost-effective microgreens.

## Data Availability Statement

The original contributions presented in the study are included in the article/Supplementary Material, further inquiries can be directed to the corresponding author/s.

## Author Contributions

Priti, MT, and AS conducted formal analysis, methodology, data curation, and writing of the original draft. GM, HD, and TS conducted conceptualization, funding acquisition, project administration, resources, writing, review and editing of the manuscript. VT, SS, MA, RK, and KT conducted methodology, writing, review and editing of the manuscript. SK, RMN, and SP conducted conceptualization, resources, writing, review and editing of the manuscript. All authors contributed to the article and approved the submitted version.

## Conflict of Interest

The authors declare that the research was conducted in the absence of any commercial or financial relationships that could be construed as a potential conflict of interest.

## Publisher's Note

All claims expressed in this article are solely those of the authors and do not necessarily represent those of their affiliated organizations, or those of the publisher, the editors and the reviewers. Any product that may be evaluated in this article, or claim that may be made by its manufacturer, is not guaranteed or endorsed by the publisher.
