# Supplementary material for: Diversity in Phytochemical Composition, Antioxidant Capacities, and Nutrient Contents Among Mungbean and Lentil Microgreens When Grown at Plain-Altitude Region (Delhi) and High-Altitude Region (Leh-Ladakh), India
Source: Front Plant Sci. 2021 Jul 30;12:710812. doi: 10.3389/fpls.2021.710812 (PMC8420906; doi:10.3389/fpls.2021.710812)
Supplement: Supplementary file 1 [file Data_Sheet_1.docx]

**Table S1. Temperature and RH in the Greenhouse of Leh and Delhi during microgreens growing duration.**

| **Growing period** | **Temperature (**ºC**)** | | **RH** | | **Sunshine**  **(h)** |
| --- | --- | --- | --- | --- | --- |
|  | **Max** | **Min** | **Max** | **Min** |  |
| Growing period (Leh, greenhouse) | 30.3±3.6 | 14.1±2.1 | 95.6±4.2 | 50.4±7.7 | 10.4±0.007 |
| Growing period (Delhi, Lentil greenhouse) | 21±1.3 | 18±1.2 | 95±2.6 | 90±1.8 | 10.3±0.005 |
| Growing period (Delhi, Mungbean greenhouse) | 28±1.1 | 26±0.9 | 95±3.2 | 90±2.4 | 10.3±0.005 |

Values represented as mean±SD (n=3)

**Table S2. Photosynthetically active radiation (PAR) and UV radiation in the greenhouse of Leh and Delhi during microgreens growing duration.**

| **Time (h)** | **PAR (μmol/m^2^s)** | | **UV A (mW/cm^2^)** | | **UV B (µW/cm^2^)** | |
| --- | --- | --- | --- | --- | --- | --- |
|  | **Leh**  **(Greenhouse)** | **Delhi**  **(Greenhouse)** | **Leh**  **(Greenhouse)** | **Delhi**  **(Greenhouse)** | **Leh**  **(Greenhouse)** | **Delhi**  **(Greenhouse)** |
| 10:00 | 624.6±21.3 | 403.4±12.5 | 0.01±0.00 | ND | 0.02±0.001 | ND |
| 12:00 | 809.3±18.8 | 509.5±17.3 | **0.02±0.00** | ND | **0.03±0.002** | ND |
| 14:00 | 769.0±10.5 | 443.2±18.2 | 0.02±0.00 | ND | 0.02±0.001 | ND |
| 16:00 | 601.1±26.1 | 350.8±21.1 | 0.01±0.00 | ND | 0.02±0.001 | ND |

Values represented as mean±SD (n=3); ND: Not detected

**Table S3. Details of the lentil and mungbean genotypes used in the study.**

| **S. No.** | **Genotype (Mungbean)** | **Collection site**  **(India)** | **Latitude /**  **Longitude** | **Average temperature**  **range (°C)** | **Rainfall (mm)** | **Variety/**  **breeding line** | **Name of institute (Developer)** |
| --- | --- | --- | --- | --- | --- | --- | --- |
|  | Pusa Baisakhi | IARI, New Delhi | 28.6329° N, 77.1449° E | 13.5-33.0 | 700.0 | Variety | IARI, New Delhi |
|  | PusaRatna | IARI, New Delhi | 28.6329° N, 77.1449° E | 13.5-33.0 | 700.0 | Variety | IARI, New Delhi |
|  | Pusa Vishal | IARI, New Delhi | 28.6329° N, 77.1449° E | 13.5-33.0 | 700.0 | Variety | IARI, New Delhi |
|  | Pusa105 | IARI, New Delhi | 28.6329° N, 77.1449° E | 13.5-33.0 | 700.0 | Variety | IARI, New Delhi |
|  | Pusa0672 | IARI, New Delhi | 28.6329° N, 77.1449° E | 13.5-33.0 | 700.0 | Variety | IARI, New Delhi |
|  | Pusa9072 | IARI, New Delhi | 28.6329° N, 77.1449° E | 13.5-33.0 | 700.0 | Variety | IARI, New Delhi |
|  | Pusa9531 | IARI, New Delhi | 28.6329° N, 77.1449° E | 13.5-33.0 | 700.0 | Variety | IARI, New Delhi |
|  | MH96-1 | CCSHAU, Hissar | 29.1504° N, 75.7057° E | 13.3 – 34.1 | 429.0 | Breeding line | CCSHAU, Hissar |
|  | MH318 | CCSHAU, Hissar | 29.1504° N, 75.7057° E | 13.3 – 34.1 | 429.0 | Variety | CCSHAU, Hissar |
|  | MH421 | CCSHAU, Hissar | 29.1504° N, 75.7057° E | 13.3 – 34.1 | 429.0 | Variety | CCSHAU, Hissar |
|  | MH521 | CCSHAU, Hissar | 29.1504° N, 75.7057° E | 13.3 – 34.1 | 429.0 | Breeding line | CCSHAU, Hissar |
|  | MH810 | CCSHAU, Hissar | 29.1504° N, 75.7057° E | 13.3 – 34.1 | 429.0 | Breeding line | CCSHAU, Hissar |
|  | ML512 | PAU, Ludhiana | 30.9010° N, 75.8071° E | 11.9-32.4 | 876.0 | Breeding line | PAU, Ludhiana |
|  | ML818 | PAU, Ludhiana | 30.9010° N, 75.8071° E | 11.9-32.4 | 876.0 | Variety | PAU, Ludhiana |
|  | PS16 | IARI, New Delhi | 28.6329° N, 77.1449° E | 13.5-33.0 | 700.0 | Variety | IARI, New Delhi |
|  | TM96-2 | BARC, Mumbai | 19.0222° N, 72.9269° E | 23.9 – 29.0 | 2012.0 | Variety | BARC, Mumbai |
|  | IPM02-3 | IIPR, Kanpur | 20.2700° N, 80.1400° E | 15.0 – 33.3 | 939.0 | Variety | IIPR, Kanpur |
|  | IPM02-14 | IIPR, Kanpur | 20.2700° N, 80.1400° E | 15.0 – 33.3 | 939.0 | Variety | IIPR, Kanpur |
|  | IPM409-4 | IIPR, Kanpur | 20.2700° N, 80.1400° E | 15.0 – 33.3 | 939.0 | Variety | IIPR, Kanpur |
|  | PMR-1 | IARI, New Delhi | 28.6329° N, 77.1449° E | 13.5-33.0 | 700.0 | Variety | IARI, New Delhi |
| **S. No.** | **Genotype**  **(Lentil)** | **Collection site**  **(India)** | **Latitude /**  **Longitude** | **Average temperature range (°C)** | **Rainfall (mm)** | **Variety/**  **breeding line** | **Name of institute**  **(Developer)** |
|  | L4076 | IARI, New Delhi | 28.6329° N, 77.1449° E | 13.5-33.0 | 700.0 | Variety | IARI, New Delhi |
|  | L4147 | IARI, New Delhi | 28.6329° N, 77.1449° E | 13.5-33.0 | 700.0 | Variety | IARI, New Delhi |
|  | L4594 | IARI, New Delhi | 28.6329° N, 77.1449° E | 13.5-33.0 | 700.0 | Variety | IARI, New Delhi |
|  | L7903 | IARI, New Delhi | 28.6329° N, 77.1449° E | 13.5-33.0 | 700.0 | Breeding line | IARI, New Delhi |
|  | HM1 | CCSHAU, Hissar | 29.1504° N, 75.7057° E | 13.3 – 34.1 | 429.0 | Variety | CCSHAU, Hissar |
|  | BM4 | BARI, Bangladesh | 23.9917° N, 90.4137° E | 18.5 – 28.3 | 507.0 | Variety | BARI, Bangladesh |
|  | JL1 | JNKVV, Jabalpur | 23.2072° N, 79.9540° E | 17.6 – 34.3 | 1208.0 | Variety | JNKVV, Jabalpur |
|  | Sehore74-3 | JNKVV, Jabalpur | 23.2072° N, 79.9540° E | 17.6 – 34.3 | 1208.0 | Variety | JNKVV, Jabalpur |
|  | NDL-1 | ANDUAT, Ayodhya | 26.5412° N, 81.8320° E | 15.2 – 32.1 | 1135.0 | Variety | ANDUAT, Ayodhya |
|  | IPL81 | IIPR, Kanpur | 20.2700° N, 80.1400° E | 15.0 – 33.3 | 939.0 | Variety | IIPR, Kanpur |
|  | IPL321 | IIPR, Kanpur | 20.2700° N, 80.1400° E | 15.0 – 33.3 | 939.0 | Variety | IIPR, Kanpur |
|  | K75 | CSAUAT, Kanpur | 26.4912° N, 80.3071° E | 15.0 – 33.3 | 939.0 | Variety | CSAUAT, Kanpur |
|  | KLS218 | CSAUAT, Kanpur | 26.4912° N, 80.3071° E | 15.0 – 33.3 | 939.0 | Variety | CSAUAT, Kanpur |
|  | DPL58 | IIPR, Kanpur | 20.2700° N, 80.1400° E | 15.0 – 33.3 | 939.0 | Breeding line | IIPR, Kanpur |
|  | DPL62 | IIPR, Kanpur | 20.2700° N, 80.1400° E | 15.0 – 33.3 | 939.0 | Variety | IIPR, Kanpur |
|  | PL1 | GBPUAT, Pantnagar | 29.0229° N, 79.4879° E | 5.4 - 20.3 | 1500.0 | Breeding line | GBPUAT, Pantnagar |
|  | PL2 | GBPUAT, Pantnagar | 29.0229° N, 79.4879° E | 5.4 - 20.3 | 1500.0 | Breeding line | GBPUAT, Pantnagar |
|  | PL6 | GBPUAT, Pantnagar | 29.0229° N, 79.4879° E | 5.4 - 20.3 | 1500.0 | Variety | GBPUAT, Pantnagar |
|  | L830 | IARI, New Delhi | 28.6329° N, 77.1449° E | 13.5-33.0 | 700.0 | Variety | IARI, New Delhi |
|  | L4602 | IARI, New Delhi | 28.6329° N, 77.1449° E | 13.5-33.0 | 700.0 | Breeding line | IARI, New Delhi |

Where, ANDUAT: Acharya Narendra Deva University of Agriculture & Technology, Ayodhya; BARI: Bangladesh Agriculture Research Institute, Bangladesh; CASUAT: Chandra Shekhar Azad University Of Agriculture & Technology, Kanpur; CCSHAU: Chaudhary Charan Singh Haryana Agricultural University, Hissar; GBPUAT: G.B.​Pant University of Agriculture & Technology, Pantnagar; IARI: Indian Agricultural Research Institute, New Delhi; IIPR: Indian Institute of Pulse Research, Kanpur; JNKVV: Jawaharlal Nehru Krishi Vishwavidyalaya, Jabalpur; PAU: Punjab Agricultural University, Ludhiana. (Source: https://www.besttimetovisit.co.in/; https://en.climate-data.org/)
